# Novel LncRNA Gm44763 Regulates Morphine-Induced Reward Memory via MiR-298-5p-Mediated eIF4E Translation Control

**DOI:** 10.34133/research.1032

**Published:** 2026-01-08

**Authors:** Feifei Gao, Xixi Yang, Zhuojin Yang, Dongyu Yu, Bao Zhang, Yihan Wang, Zhen Yao, Jie Chen, Qi Liao, Lanjiang Li, Beilin Hou, Danmei Wang, Yuxiang Zhang, Chunxia Yan

**Affiliations:** ^1^ College of Forensic Medicine, NHC Key Laboratory of Forensic Medicine, Xi’an Jiaotong University Health Science Center, Xi’an 710061, Shaanxi, China.; ^2^ Bio-evidence Science Academy, Science and Technology Innovation Harbour of Western China, Xi’an Jiaotong University, Xi-xian New Area Fengxi New City 712061, Shaanxi, China.; ^3^ The First Clinical Medical College, Xi’an Jiaotong University Health Science Center, Xi’an 710061, Shaanxi, China.; ^4^ National Narcotics Laboratory Shaanxi Branch, Xi-xian New Area Fengxi New City 712000, Shaanxi, China.; ^5^ School of Forensic Medicine, Kunming Medical University, NHC Key Laboratory of Drug Addiction Medicine, Kunming 650504, Yunnan, China.

## Abstract

Drug-associated reward memory underlies both the development and relapse of addiction, yet its molecular basis remains poorly understood. Here, transcriptomic profiling and functional validation identified a novel long non-coding RNA (lncRNA), Gm44763, as a critical regulator of morphine-induced reward memory specifically in neurons of the medial prefrontal cortex (mPFC). Behavioral and molecular analyses demonstrated that Gm44763 functions as a sponge for miR-298-5p, thereby relieving the repression of the downstream target gene, eukaryotic translation initiation factor 4E (eIF4E), and modulating both the acquisition and retrieval of reward memory. Golgi staining and fiber photometry further revealed that Gm44763 normalized morphine-induced alterations in synaptic structure and neuronal excitability. miR-298-5p bidirectionally regulated morphine-induced reward memory and reversed both behavioral and neuronal effects mediated by Gm44763. Mechanistically, the downstream effector eIF4E modulates translation via its interaction with eIF4G, thereby contributing to morphine-induced memory regulation. This process can be effectively modulated by 4EGI-1, a selective inhibitor of the eIF4E/eIF4G interaction. In summary, this study characterized lncRNA expression profiles in the mPFC of mice with morphine-induced conditioned place preference. We identified and validated Gm44763 as a novel lncRNA regulator of morphine-induced reward memory and synaptic plasticity. We further delineate a previously uncharacterized Gm44763/miR-298-5p/eIF4E axis that may represent a novel regulatory pathway linking transcriptional and translational control to drug-associated memory formation.

## Introduction

Addiction represents a persistent brain disorder marked by compulsive drug seeking and a pronounced vulnerability to relapse. Substantial evidence indicates that the formation and retrieval of drug-associated reward memories are central mechanisms driving the development and recurrence of addictive behaviors. The medial prefrontal cortex (mPFC) orchestrates executive, emotional, and mnemonic functions, enabling the integration of environmental information with motivational drive, thereby contributing to drug-seeking behavior and the maintenance of reward memories [1,2]. Repeated exposure to addictive substances induces adaptive changes in synaptic structure and function within the mPFC, including alterations in dendritic spine morphology and synaptic transmission, which together promote the persistence of addictive behaviors. Although previous studies have primarily focused on classical neurotransmitter systems such as dopamine and glutamate, as well as receptor-mediated signaling pathways [3,4], accumulating evidence suggests that epigenetic and post-transcriptional regulation of gene expression is critical for sustaining drug-associated memories [5,6]. Among these regulatory mechanisms, non-coding RNAs, especially long non-coding RNAs (lncRNAs), have emerged as important modulators of synaptic plasticity, memory, and various neuropsychiatric disorders [7]. However, the specific roles of lncRNAs in the formation and retrieval of drug-associated reward memories remain largely unexplored.

LncRNAs comprise a class of transcripts over 200 nucleotides in length that lack protein-coding capacity but exert important regulatory roles in gene expression [8]. Extensive evidence has demonstrated that lncRNAs participate in key neural functions, such as neuronal development, regulation of synaptic plasticity, and mechanisms of learning and memory [7]. Previous studies have identified distinct lncRNA expression profiles associated with addiction. For example, marked alterations in lncRNA expression have been observed in the midbrains of human cocaine users [9]. Similarly, cocaine-induced conditioned place preference (CPP) in mice leads to dysregulated lncRNA expression in the nucleus accumbens (NAc) [10]. Differentially expressed lncRNAs (DElncRNAs) have also been identified in the NAc of mice subjected to morphine-induced CPP [11]. However, the lncRNA expression profiles in the mPFC of mice following morphine-induced CPP have not yet been elucidated. Mechanistically, lncRNAs function as competing endogenous RNAs (ceRNAs), sequestering microRNAs (miRNAs) to modulate downstream gene expression [12–14]. LncRNA-mediated ceRNA mechanisms have been increasingly recognized as pivotal regulators of neuronal plasticity, cognitive functions, and the development of neuropsychiatric disorders. Nevertheless, the functional involvement of lncRNA-mediated ceRNA networks in the formation and retrieval of drug-associated reward memories, particularly in the context of morphine addiction, remains largely unexplored.

Synaptic plasticity, defined as the structural and functional remodeling of synapses, underlies core cognitive processes including learning, memory formation, and adaptive responses [15]. The cap-binding protein eukaryotic translation initiation factor 4E (eIF4E) is indispensable for translation initiation, mediating ribosome recruitment to capped mRNAs, a mechanism essential for plastic changes at synapses and the consolidation of memory [16]. Specifically, the assembly of the eIF4E–eIF4G complex into eIF4F constitutes a rate-limiting step in translation initiation, and this process is modulated by addiction-related pathways including mammalian target of rapamycin (mTOR) and extracellular signal-regulated protein kinase (ERK) [17,18]. Accumulating evidence indicates that eIF4E activity undergoes dynamic changes following exposure to drugs of abuse. For example, cocaine administration substantially increases phosphorylation of eIF4E at Ser209 in the ventral tegmental area, which correlates with enhanced translation of synaptic proteins and increased CPP behavior [19]. Chronic alcohol exposure has been shown to reduce eIF4E–eIF4G interaction in the rat heart [20], suggesting tissue- and context-specific translational dysregulation. Moreover, pharmacological disruption of eIF4E–eIF4G interaction rescues synaptic dysfunction and autism-like behaviors in mouse models [18]. The critical role of eIF4E–eIF4G interaction in hippocampal synaptic plasticity has also been well established [21], highlighting its importance in neurodevelopmental and neuroplasticity-related disorders. However, the role of eIF4E-mediated translational control in opioid-induced synaptic remodeling remains incompletely understood.

In this study, we identified and validated a novel lncRNA, Gm44763, as a key regulator of morphine-induced reward memory. We hypothesized that Gm44763 functions as a ceRNA to regulate the expression of eIF4E by sponging miR-298-5p, thereby influencing synaptic plasticity and behavioral outcomes. By integrating transcriptomic analysis, behavioral paradigms, in vivo calcium imaging, molecular assays, and translational regulation analyses, we systematically investigated the Gm44763/miR-298-5p/eIF4E axis in the mPFC. Our findings uncover a previously unrecognized regulatory network linking Gm44763-mediated ceRNA mechanisms to translational control and the modulation of drug-associated memory. This work deepens mechanistic insights into addiction-related memory and identifies potential molecular targets for innovative treatments of substance use disorders.

## Results

### Establishment of morphine-induced CPP model and identification of DElncRNAs in mice

**Fig. 1.**
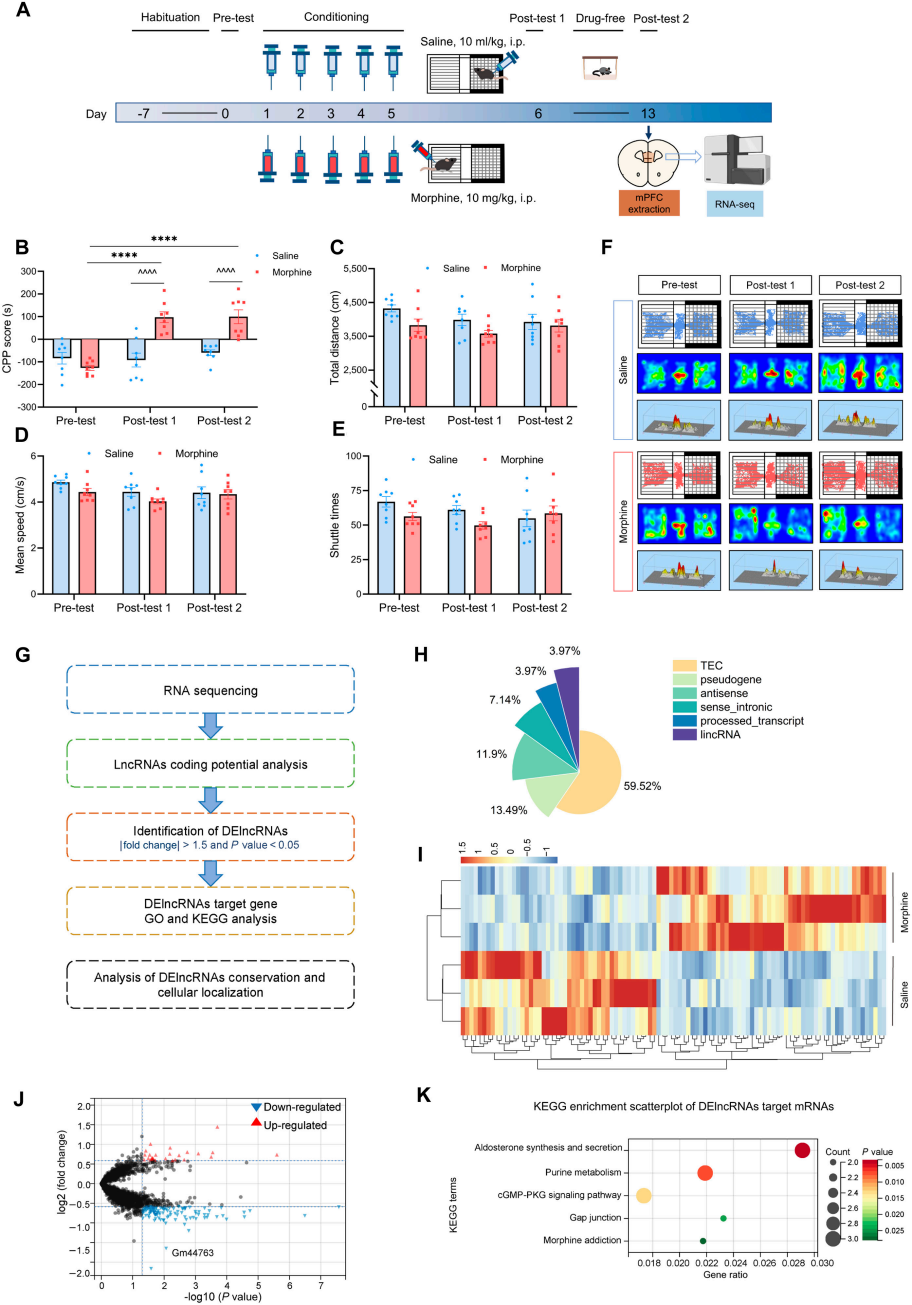
Morphine-induced CPP model establishment in mice after 5 rounds of training and identification of differentially expressed lncRNAs. (A) Experimental schedule for morphine-induced CPP. Mice first underwent a 7-day habituation period, then underwent pre-test on day 0 and exposed to 5 days of consecutive training (days 1 to 5). From days 7 to 12, mice were housed in the home cage without drug administration. The retrieval test was performed on day 13 and mPFC tissue was extracted for RNA sequencing. (B) CPP scores of pre-test (day 0), post-test 1 (day 6), and post-test 2 (day 13). Data are expressed as mean ± SEM (*n* = 8). ****P < 0.0001 vs. pre-test; ^^^^^^*P* < 0.0001 vs. saline group (2-way ANOVA followed by Bonferroni post hoc test). (C to E) Moving distance, mean speed, and shuttle times of pre-test, post-test 1 and post-test 2. ****P < 0.0001 vs. pre-test; ^^^^*P* < 0.0001 vs. saline group. *n* = 8 mice/group. (F) Representative motor paths and heatmap images of mice from both groups during the tests. The white chamber on the left represents the morphine-paired compartment. (G) Flowchart for validating and analyzing DElncRNAs. (H) Proportion of different types of lncRNAs among the identified DElncRNAs. (I) Heatmap showing clusters of top 100 DElncRNAs between saline and morphine samples. Red color gradients correspond to positive fold-change values, whereas blue gradients correspond to negative values. (J) Volcano plot illustrating DElncRNAs. Up-regulated lncRNAs are marked in red, while down-regulated ones are marked in blue. (K) KEGG enrichment analysis of DEmRNAs revealed significant enrichment in the cGMP-PKG signaling pathway and morphine addiction.

The experimental procedure for morphine-induced CPP is shown in Fig. 1A. Mice were administered saline (10 ml/kg) or morphine (10 mg/kg) and assessed for CPP scores during the pre-test, post-test 1, and post-test 2. Compared with saline controls, morphine exposure markedly elevated CPP scores in post-test 1 and post-test 2, with no baseline difference observed at pre-test (Fig. 1B). Additionally, we measured the total distance, mean speed, and shuttle times. No significant variations were identified between the groups, suggesting that morphine-induced reward memory does not affect exploratory behavior or locomotor performance in mice (Fig. 1C to E). Representative motor paths and heatmap images of mice from both groups in the tests are shown in Fig. 1F.

According to the threshold criteria shown in Fig. 1G, we identified 126 DElncRNAs, including 22 that were up-regulated and 104 that were down-regulated. The distribution of DElncRNAs is presented in Fig. 1H. To further explore expression patterns, the top 100 DElncRNAs were selected to generate a heatmap, which revealed distinct clustering of morphine-exposed samples and saline controls (Fig. 1I). The volcano plot (Fig. 1J) depicts down-regulated lncRNAs in blue and up-regulated lncRNAs in red. Table S1 summarizes the top 10 up-regulated and down-regulated DElncRNAs, along with their corresponding fold changes.

Moreover, lncRNAs regulate nearby mRNAs [22], so we employed an optimized version of the strategy reported by Ye et al.[23], analyzing genes situated within 100 kb upstream or downstream of DElncRNAs as putative targets [24]. Based on the previously generated mRNA sequencing dataset from the same experimental model in our lab (GSE221797), we defined differentially expressed mRNAs (DEmRNAs) using thresholds of |fold change| > 2 and *P* < 0.05. Target mRNAs that overlapped with these DEmRNAs were selected for Gene Ontology (GO) and Kyoto Encyclopedia of Genes and Genomes (KEGG) pathway enrichment analyses. GO enrichment results highlighted terms related to synaptic remodeling and neuronal functions (Fig. S1). Consistently, KEGG pathway analysis revealed significant involvement of the cGMP-PKG signaling pathway (mmu04022), previously linked to cognitive and memory processes [25]. Notably, morphine addiction (mmu05032) was also significantly enriched (Fig. 1K). These findings suggest that DEmRNAs in the mPFC may contribute to morphine-induced reward memory.

### Screening and identification of Gm44763 as a candidate lncRNA regulating morphine-induced reward memory

**Fig. 2.**
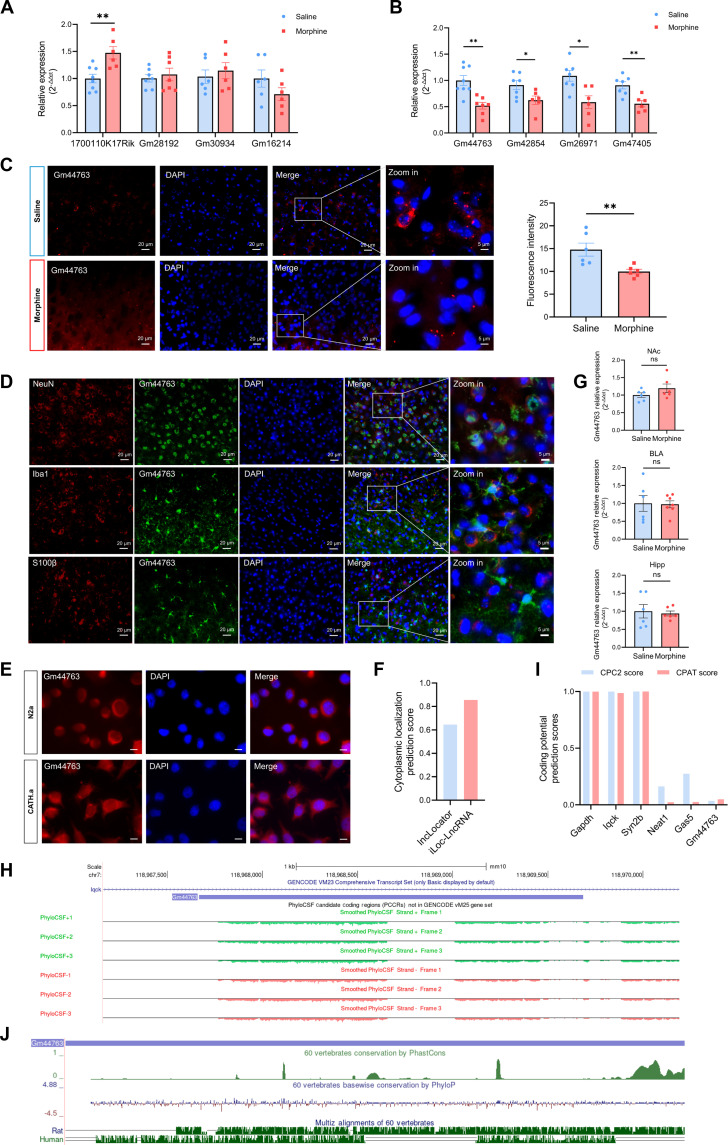
Gm44763 expression is reduced in the mPFC of mice with morphine-induced CPP. (A) The qPCR analysis of 4 up-regulated lncRNAs randomly selected from the sequencing data. Among these, 1700110k17rik was significantly up-regulated, while Gm28192and Gm30934 showed an upward trend. n = 6 to 8. *P < 0.05, Student’s t test, compared with the saline group. (B) qPCR analysis of 4 down-regulated lncRNAs randomly selected from the sequencing data. Gm44763, Gm42854, Gm26971, and Gm47405 were significantly down-regulated. *n* = 6 to 8, *P < 0.05, **P < 0.01 compared with the saline group. Student’s *t* test. (C) RNA FISH assays confirmed that the expression of Gm44763 in mPFC of morphine-treated mice were significantly lower than in the saline group. *n* = 6. **P < 0.01, Student’s *t* test. (D) Representative images show the colocalization of Gm44763 (red) with the neuronal marker NeuN (green), the microglial marker Iba1 (green), and the astrocytic marker S100β (green). Subcellular localization revealed that Gm44763 surrounds NeuN-positive nuclei and is distributed within the cytoplasm, while exhibiting no overlap with Iba1 or S100β. (E) RNA FISH assays were performed to analyze the subcellular distribution of Gm44763 in N2a and CATH.a cells; scale bar = 10 μm. (F) lncLocator and iLoc-LncRNA predictions supported the cytoplasmic enrichment of Gm44763. (G) Regional expression analysis revealed that Gm44763 showed no significant changes in the NAc, BLA, or Hipp of mice following morphine-induced CPP. *n* = 6. (H) Chromosomal location of Gm44763 and PhyloCSF scores for this region. (I) The CPAT and CPC scores of Gm44763 were significantly lower than those of the coding transcripts (Syn2a, Iqck, and Gapdh) as with known lncRNAs (Neat1 and Gas5). (J) Evolutionary conservation across 60 vertebrate species was assessed using PHAST package, and comparative genomic alignments between mouse, rat, and human genomes were visualized.

To validate the reliability of the differential expression profiles identified by RNA sequencing (RNA-seq), we randomly selected 4 DElncRNAs from the top 10 up-regulated (Fig. 2A) and top 10 down-regulated (Fig. 2B) candidates for validation by quantitative real-time polymerase chain reaction (qPCR) in mouse mPFC samples. As expected, the qPCR results were largely consistent with the RNA-seq findings, with Gm44763 showing the most pronounced change in expression. Moreover, fluorescence in situ hybridization (FISH) analysis further confirmed a significant down-regulation of Gm44763 following morphine-induced CPP (Fig. 2C).

To determine the cellular localization, FISH combined with immunofluorescence staining revealed that Gm44763 was predominantly expressed in the cytoplasm of neurons, with only minimal signals in microglia and astrocytes (Fig. 2D). This indicates that Gm44763 is primarily expressed and functions within neurons. We also performed FISH in 2 mouse neuronal cell lines, N2a and CATH.a, and confirmed that Gm44763 is predominantly localized in the cytoplasm (Fig. 2E). In addition, the subcellular localization of Gm44763 was predicted from its sequence using online analysis tools. The cytoplasmic localization score was 0.645 as predicted by lncLocator [26], and 0.855 as predicted by iLoc-LncRNA [27], both supporting cytoplasmic enrichment (Fig. 2F). Furthermore, we found that morphine-induced CPP selectively altered Gm44763 expression in the mPFC, but not in the NAc, basolateral amygdala (BLA), or hippocampus (Hipp) (Fig. 2G). These findings indicate that Gm44763 is a spatially and cell-type-specific lncRNA associated with addiction.

Gm44763 is located on the negative strand of chromosome 7 and consists of a single exon of 2,019 bp, according to the UCSC (https://genome.ucsc.edu/index.html) and ensembl (http://asia.ensembl.org/index.html) databases (Fig. 2H). To confirm that Gm44763 is an lncRNA, we employed the PhyloCSF coding potential analysis method of open reading frame (ORF) and exon comparison provided by the UCSC database. The PhyloCSF values for the region containing Gm44763 were all negative, indicating that it is a non-coding region (Fig. 2H). We further assessed its coding potential using the Coding Potential Assessment Tool (CPAT) [28] and Coding Potential Calculator (CPC) [29]. The results show that Gm44763 scored very low in both algorithms compared to known protein-coding transcripts, similar to well-known lncRNAs (e.g., Neat1 and Gas5) (Fig. 2I).

We next assessed whether Gm44763 is evolutionarily conserved across species. Two algorithms from the PHAST package, phastCons and phyloP, were accessed via the UCSC Genome Browser to evaluate sequence conservation across 60 vertebrate species [30]. A summary alignment between the mouse genome and other species, including rat and human, was visualized. Densely clustered green bars indicated regions of high sequence conservation (Fig. 2J). The results indicate that Gm44763 exhibits a relatively high degree of evolutionary conservation across species. Therefore, Gm44763 was selected as a candidate lncRNA for subsequent analysis of its function in morphine-induced reward memory.

### Overexpression of Gm44763 in the mPFC impairs morphine-induced reward memory

**Fig. 3.**
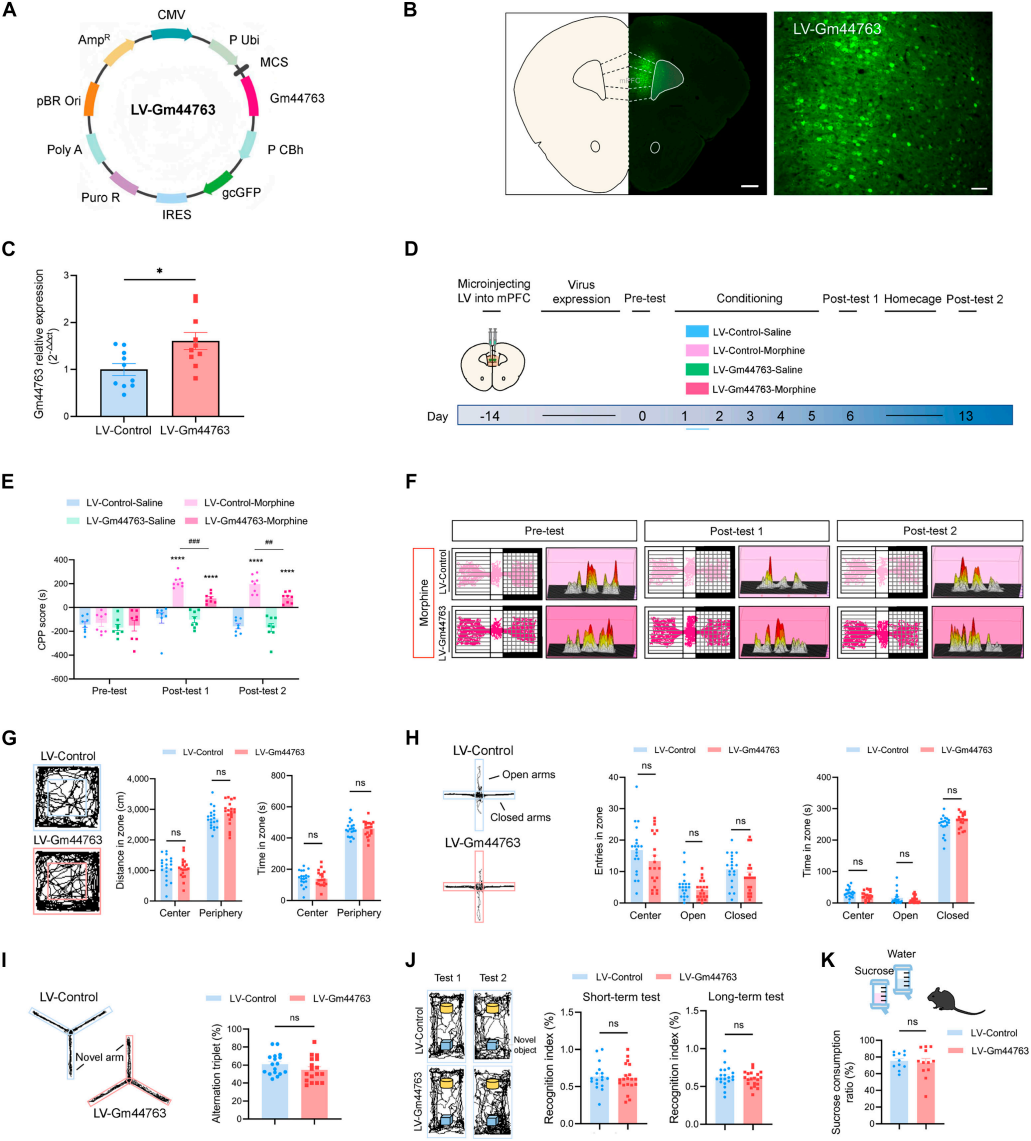
Overexpression of Gm44763 in the mPFC attenuated the rewarding effects of morphine. (A) Schematic representation of LV-Gm44763 constructs. (B) (Left) Fluorescence microscopy images demonstrate localized GFP expression in the mPFC. Scale bar = 500 μm. (Right) Fluorescence verification of mPFC-specific transduction. Scale bar = 50 μm. (C) qPCR validation of Gm44763 overexpression efficiency in the mPFC. *n* = 10, *P < 0.05 (Student’s *t* test). (D) Schematic timeline of the experimental procedure. LV-Gm44763 or LV-Control was stereotactically injected into the mPFC 14 days before CPP pre-test (day 0), followed by morphine/saline conditioning (days 1 to 5) and post-test 1 (day 6). Post-test 2 was conducted on day 13, after which mPFC tissue was collected for molecular analyses. (E) During both post-test 1 and post-test 2 phases, the CPP scores of the LV-Gm44763 + Morphine group were significantly lower than those of the LV-Control + Morphine group. Two-way ANOVA followed by Bonferroni post hoc test, ****P < 0.0001 vs. pre-test score of the same group; ##*P* = 0.006, ###*P* = 0.0008, morphine-induced differences in the LV-Gm44763 group of mice vs. LV-Control group in the post-tests, *n* = 8. (F) Representative locomotor traces and heatmap images of mice from the LV-Gm44763 and LV-Control groups during the CPP procedure following morphine conditioning. The white chamber on the left represents the morphine-paired compartment. (G) (Left) Representative locomotor activity traces in the OFT. (Right) No significant differences were observed between the LV-Gm44763 and control groups in total distance traveled or time spent in the center and periphery zones. *n* = 20, Student’s *t* test. (H) (Left) Representative locomotor activity traces in the EPM. (Right) No significant differences between LV-Gm44763 and control groups in either the number of arm entries or the time spent in open and closed arms. *n* = 20, Student’s *t* test. (I) (Left) Representative locomotor activity traces in the Y-maze. (Right) No significant difference in the alternation triplet in the LV-Gm44763 group of mice compared with the LV-Control group of mice. *n* = 17, Student’s *t* test. (J) (Left) Representative locomotor activity traces in the NOR. (Right) No significant differences were observed in the recognition index for either the short-term memory test (test 1) or the long-term memory test (test 2) between LV-Gm44763 and LV-Control mice. *n* = 17 to 20, Student’s *t* test. (K) In the sucrose preference test (SPT), mice showed a natural preference for sucrose, consuming more than 70% sucrose. *n* = 11 to 12, Student’s *t* test.

To investigate the causal role of Gm44763 in morphine-induced CPP in mice, we used lentivirus (LV) overexpressing Gm44763 (LV-Gm44763) and an empty vector (LV-Control) as a control (Fig. 3A). LV-mediated GFP expression was restricted to the mPFC (Fig. 3B). Gm44763 expression was significantly increased in the mPFC of mice injected with LV-Gm44763, demonstrating that LV-Gm44763 induced significant overexpression of Gm44763 (Fig. 3C). As shown in Fig. 3D, the CPP test was performed after the LV was expressed. Mice in the LV-Gm44763 and LV-Control injection groups exhibited similar CPP scores in the CPP pre-test. Although all mice exhibited morphine-induced CPP, mice injected with LV-Gm44763 displayed significantly decreased CPP scores compared with LV-Control mice (Fig. 3E). Representative locomotor trajectories and corresponding heatmaps of the 2 groups of morphine-treated mice during the experiment are shown in Fig. 3F. Overexpression of Gm44763 did not affect the locomotor ability of mice, as no significant differences were observed in total distance, mean speed, and shuttle times across the groups within the CPP chambers, thereby excluding the confounding effects of locomotion on the behavioral outcomes (Fig. S2).

Following viral expression, we conducted open field test (OFT) and elevated plus maze (EPM) test to assess the effects of LV-Gm44763 on locomotor activity and anxiety-like behavior in mice. No significant differences were observed during behavioral testing (Fig. 3G and H). Y-maze and novel object recognition (NOR) tests were also performed to evaluate spatial and recognition memory. No significant differences were found between LV-Gm44763 and LV-Control groups (Fig. 3I and J).

We further investigated whether modification of Gm44763 expression in mPFC affects the preference of mice for sucrose, a measure of natural reward sensitivity. The results showed that sucrose preference remained unchanged in mice overexpressing Gm44763 compared to controls (Fig. 3K). In summary, these findings indicate that Gm44763 plays a specific regulatory role in morphine-induced reward memory.

### Gm44763 overexpression reverses morphine-induced synaptic remodeling and neuronal hyperactivity

**Fig. 4.**
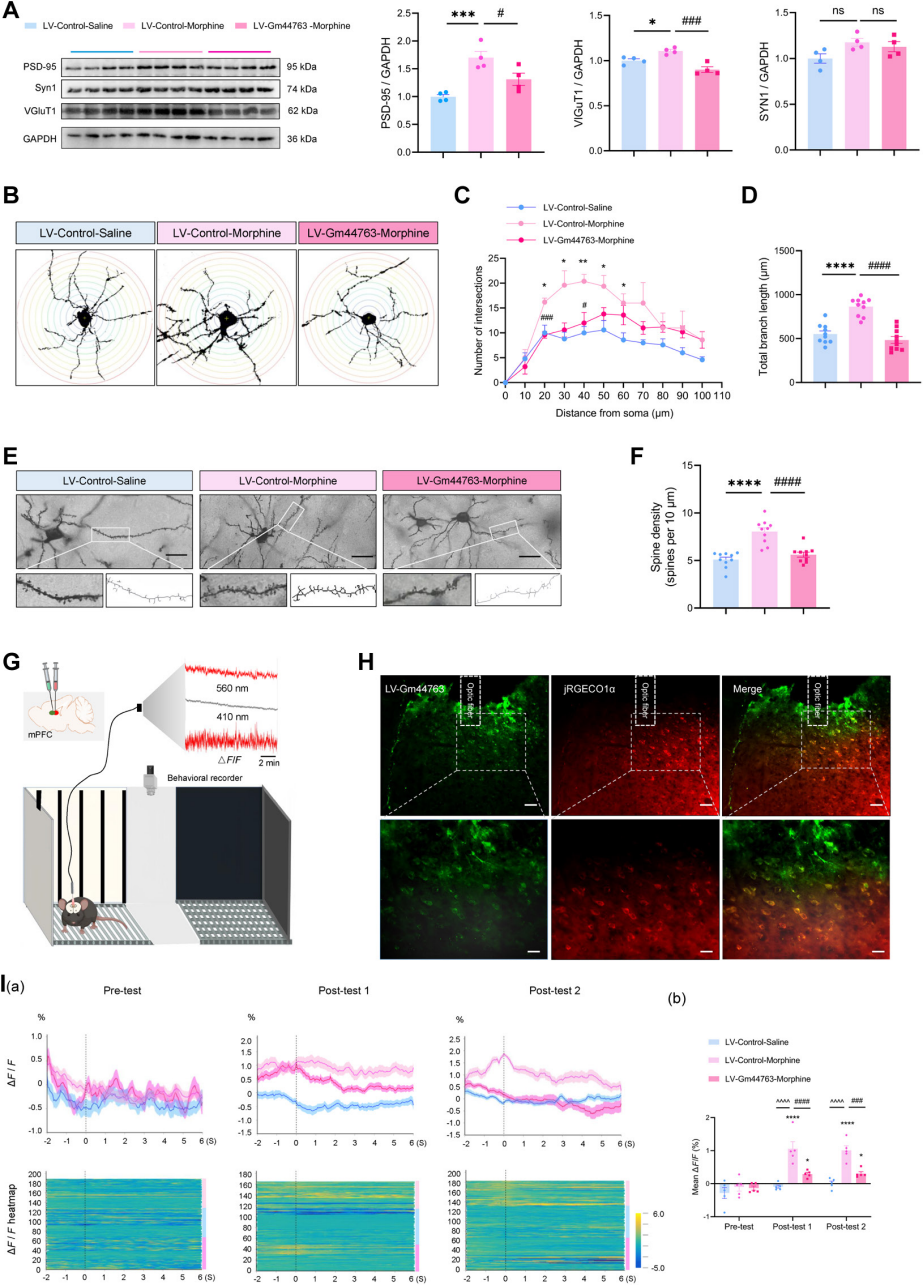
Gm44763 overexpression reverses morphine-induced synaptic remodeling and neuronal hyperactivity. (A) Synaptic protein dysregulation (PSD-95, VGluT1) in morphine-induced mice was rescued by Gm44763 overexpression. Syn1 protein levels showed no significant variation across groups. *n* = 4. *P < 0.05, ***P < 0.001, compared with LV-Control-Saline; #*P* < 0.05, ###*P* < 0.001 compared with LV-Control-Morphine. (B) Golgi–Cox staining visualized neuronal morphology in the mPFC. Sholl analysis was performed using concentric circles with 10-μm intervals and a radius range of 0 to 100 μm to quantify dendritic complexity. (C) The Sholl intersection profile revealed that morphine exposure enhanced dendritic complexity in the mPFC, which was normalized following Gm44763 overexpression. The *X*-axis represents the radial distance from the soma, while the *Y*-axis indicates the number of intersections at each concentric circle. *n* = 5. *P < 0.05, **P < 0.01 compared with LV-Control-Saline; #*P* < 0.05, ###*P* < 0.001 compared with LV-Control-Morphine. (D) Quantification of total dendritic branch length showed that morphine conditioning significantly increased branch length, while Gm44763 overexpression restored dendritic length to baseline levels. *n* = 10. ****P < 0.0001 compared with LV-Control-Saline; ####*P* < 0.0001 compared with LV-Control-Morphine. (E) Representative images of dendrites in the mPFC of mice. Scale bar = 25 μm. (F) Quantification of dendritic spine density (spines per 10 μm) showed that morphine exposure increased spine density, while Gm44763 overexpression normalized this effect. *n* = 10. ****P < 0.0001 compared with LV-Control-Saline; ####*P* < 0.0001 compared with LV-Control-Morphine. (G) Schematic illustration of the experimental setup showing coinjection of the red calcium indicator AAV-hSyn-JRGECO1a and the green LV-Gm44763 into the mPFC, followed by fiber implantation for in vivo calcium dynamics recording during the CPP test. (H) Fluorescence validation showing coexpression of the 2 viruses in the mPFC. Top: scale bar = 200 μm; bottom: scale bar = 20 μm. (I) Gm44763 overexpression partially reversed the morphine-induced neuronal hyperactivity, restoring calcium signaling toward baseline levels. (a) Calcium signals (Δ*F*/*F*) recorded from mPFC neurons of mice during pre-test, post-test 1, and post-test 2, showing calcium activity 2 s before and 6 s after entry events. Bottom: Time-aligned heatmaps of calcium signals (−2 to +6 s) relative to chamber entry. Each row represents an individual entry event. Color intensity reflects the normalized Δ*F*/*F* amplitude. (b) Quantification of the average calcium transients during chamber entry events. Overexpression of Gm44763 significantly attenuated morphine-induced neuronal hyperexcitability. *n* = 5. *P < 0.05, ****P < 0.0001 compared with pre-test; ^^^^^^*P* < 0.0001 compared with LV-Control-Saline; ###*P* < 0.001, ####*P* < 0.0001 compared with LV-Control-Morphine. Data analyzed using 2-way ANOVA with Bonferroni post hoc test.

Given the well-established association between memory and synaptic plasticity [31], we collected mPFC tissue after behavioral testing to assess the expression of synapse-related markers. Western blot analysis demonstrated a significant up-regulation of postsynaptic density protein-95 (PSD-95) and vesicular glutamate transporter 1 (VGluT1) following morphine exposure, which was reversed by Gm44763 overexpression, restoring these protein levels to baseline. However, Synapsin I (Syn1) expression did not differ significantly across the groups (Fig. 4A). These results suggest that Gm44763 may exert its effects by normalizing morphine-induced synaptic adaptations in the mPFC.

Based on the regulatory effects of Gm44763 on synapse-associated proteins, we performed neuronal morphological analysis following behavioral testing to investigate the underlying synaptic plasticity mechanisms in the mPFC. Golgi staining combined with Sholl analysis revealed that morphine exposure significantly enhanced neuronal dendritic complexity (Fig. 4B and C) and increased total dendritic branch length of mPFC neurons (Fig. 4D). Notably, overexpression of Gm44763 effectively reversed the morphine-induced dendritic structural alterations, restoring dendritic architecture to baseline levels (Fig. 4B to D). We further analyzed dendritic spine density, with representative images shown in Fig. 4E. Morphine exposure markedly elevated spine density, whereas Gm44763 overexpression normalized this change (Fig. 4F). Collectively, these findings suggest that Gm44763 may exert its regulatory effects on morphine-induced reward memory by modulating synaptic plasticity in the mPFC.

We next explored whether Gm44763 also modulates neuronal excitability in the mPFC during morphine-induced reward memory. In vivo fiber photometry was performed to monitor calcium transients in mPFC neurons. LV-Gm44763 and the calcium indicator jRGECO1a were stereotaxically coinjected into the mPFC, followed by optical fiber implantation (Fig. 4G). During the pre-test and post-tests, dual-wavelength excitation (560 nm for signal and 410 nm for reference) was applied to quantify standardized Δ*F*/*F* calcium signals in the mPFC. Fluorescence imaging confirmed the colocalization of both viral within mPFC, ensuring the specificity of the recorded signals (Fig. 4H). The results demonstrated that neuronal activity in morphine-induced CPP mice increased upon entry into the drug-paired chamber. Notably, Gm44763 overexpression partially reversed the morphine-induced neuronal hyperactivity, restoring calcium signaling toward baseline levels. No significant differences in calcium signaling were detected among groups during the pre-test (Fig. 4I), thereby excluding the potential confounding effects of viral delivery or genetic manipulation on baseline neuronal function. Based on these findings, we hypothesize that Gm44763 modulates morphine-induced reward memory by regulating synaptic remodeling and inducing alterations in neuronal excitability.

### Construction and validation of the Gm44763/miR-298-5p/eIF4E regulatory network

**Fig. 5.**
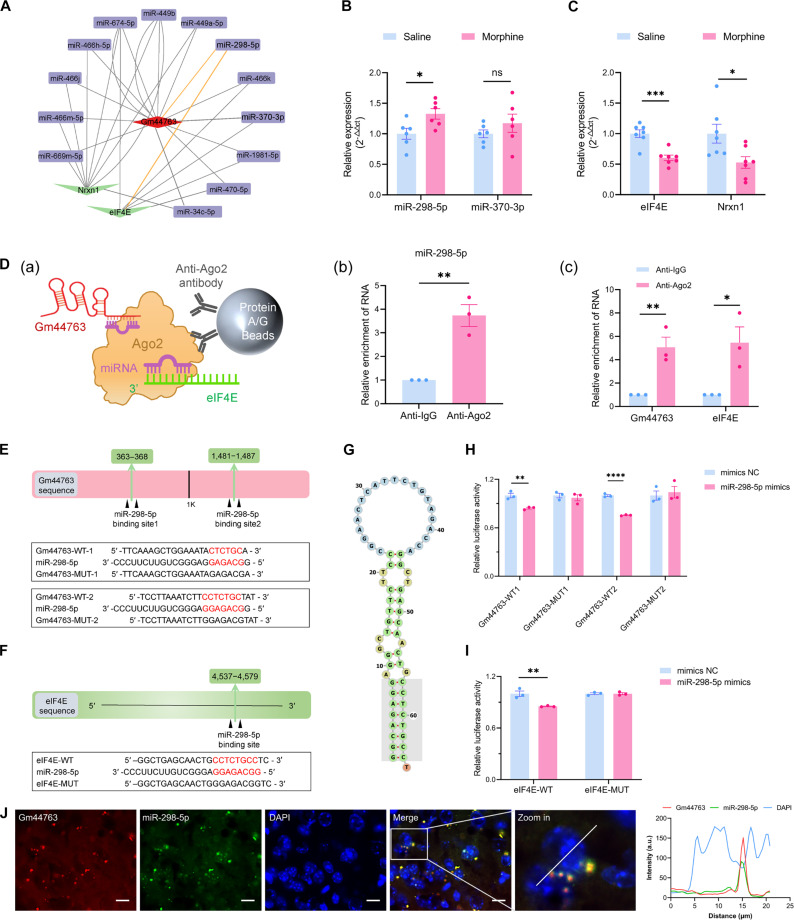
Identification and validation of the Gm44763-mediated ceRNA regulatory network. (A) Gm44763-mediated ceRNA network. In this network, red diamond represents lncRNA, purple rectangles represent miRNAs, and green ellipses represent mRNAs. (B and C) qPCR analysis showed that miR-298-5p expression was significantly up-regulated in mice following morphine exposure, while miR-370-3p expression remained unchanged. Both eIF4E and Nrxn1 were significantly down-regulated after morphine treatment. *n* = 6 to 7. *P < 0.05, ***P < 0.001 compared with the saline group. (D) (a) RIP experimental schematic of Gm44763 and eIF4E as RISC target genes. (b and c) RIP was performed on N2a cell lysates with anti-Ago2 or IgG antibodies. In the same precipitates, anti-Ago2 enriched miR-298-5p, Gm44763, and eIF4E. *n* = 3, *P < 0.05, **P < 0.01 compared with IgG-enriched group. (E) Schematic diagram of the binding site between Gm44763 and miR-298-5p. (F and G) Schematic diagram of the binding site between eIF4E and miR-298-5p, with the longest consecutive pairing consisting of 8 bases. The binding sites are indicated by red text or shaded gray areas. (H) Dual-luciferase assays verified the direct binding of Gm44763 to miR-298-5p. miR-298-5p mimics significantly reduced luciferase activity in both WT1 and WT2 reporters. Data were shown as the relative luciferase activity. *n* = 3. **P < 0.01, ****P < 0.0001 compared with mimics NC. (I) Targeted binding between eIF4E and miR-298-5p was demonstrated by dual-luciferase reporter assays. *n* = 3, **P < 0.01 compared with mimics NC group. Data analyzed using Student’s *t* test. (J) The FISH experiment showing the colocalization of Gm44763 (red) and miR-298-5p (green) in the mouse mPFC. Scale bar = 10 μm.

Given that Gm44763 is mainly localized in the cytoplasm, we first hypothesized that Gm44763 may interact with specific miRNAs to regulate morphine-induced CPP. Accumulating evidence suggests that lncRNAs and mRNAs display coexpression patterns within ceRNA networks. Therefore, we selected DEmRNAs from the Gene Expression Omnibus (GEO) database (GSE221797) that exhibited a down-regulation trend concordant with that of Gm44763 to construct the ceRNA network. To enhance the biological relevance of the ceRNA network to morphine-induced reward memory, we further prioritized DEmRNAs associated with neurological and neuropsychiatric disorders through systematic literature curation. As shown in Fig. S3A, candidate miRNAs targeting DElncRNAs were predicted using RNAhybrid and miRDB databases, while miRNA targets of DEmRNAs were identified using TargetScan, ENCORI, miRWalk, and RNAhybrid. The intersection of these datasets yielded a set of candidate miRNAs. This integrative multi-algorithm approach enabled the delineation of a Gm44763-mediated ceRNA regulatory network (Fig. 5A). Within this network, miR-298-5p has been shown to play a role in neurodegeneration and chronic stress [32]. Dysregulation of miR-370-3p has been observed in the brain tissues of bipolar disorder patients [33]. The cap-binding protein eIF4E interacts with mRNA to mediate ribosome recruitment and initiation of translation, thereby acting as a major regulator of initiation rate. It is implicated in autism and in the antidepressant actions of ketamine [34]. Neurexin 1 (Nrxn1) is crucial for synapse formation, transmission, and plasticity, and is implicated in learning, memory, and multiple neuropsychiatric disorders including schizophrenia and autism [35,36]. The expression profiles of miR-298-5p (Fig. 5B) and eIF4E (Fig. 5C) in morphine-induced CPP mice, as determined by qPCR, aligned with the ceRNA regulatory model involving Gm44763, further supporting the biological relevance of this interaction.

To validate the interaction between miR-298-5p, eIF4E, and Gm44763, we performed RNA immunoprecipitation (RIP) assays in N2a cells to determine whether Gm44763 and eIF4E serve as RNA-induced silencing complex (RISC) targets [Fig. 5D(a)]. The results demonstrated that both Gm44763 and eIF4E directly bind to Ago2 and are enriched in Ago2 immunoprecipitates [Fig. 5D(b and c)]. Subsequent bioinformatic analysis revealed that Gm44763 shares 2 complementary binding sites with miR-298-5p (Fig. 5E). Similarly, eIF4E shares one complementary binding site with miR-298-5p (Fig. 5F and G). Dual-luciferase assays were performed by inserting wild-type or mutant sequences of Gm44763 or eIF4E into the pmirGLO vector and cotransfecting them with miR-298-5p mimics or negative control (mimics NC) into 293T cells. Luciferase activity was significantly reduced by miR-298-5p mimics, whereas mutations in the binding sites abolished this effect (Fig. 5H and I). We used FISH experiments to visualize the spatial colocalization of Gm44763 and miR-298-5p (Fig. 5J). These findings suggest that Gm44763 may function through direct interaction with both miR-298-5p and eIF4E within this regulatory axis.

**Fig. 6.**
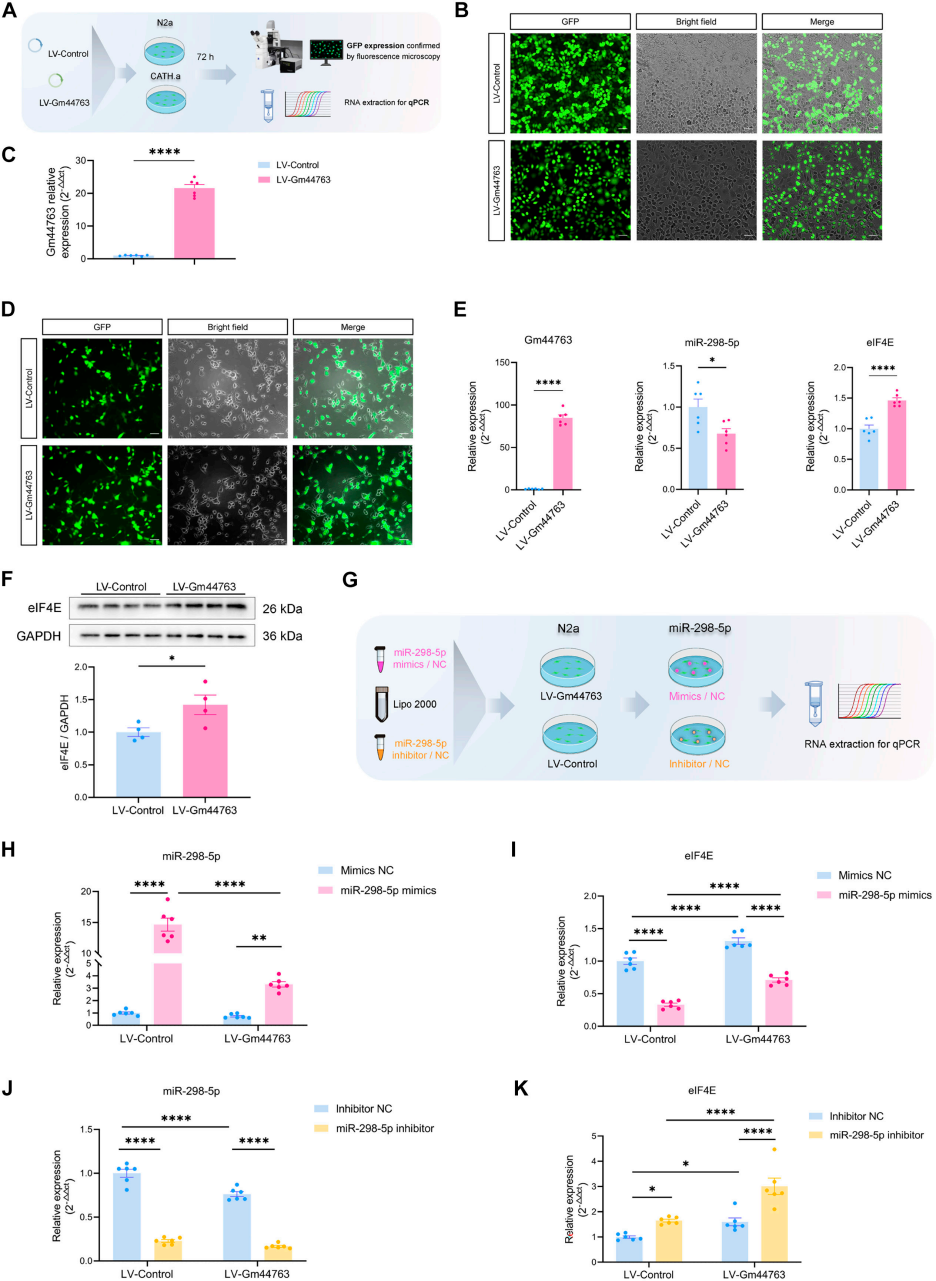
In vitro validation of Gm44763 regulation of eIF4E expression via competitive binding to miR-298-5p. (A) Experimental schedule to validate the efficacy of overexpression in LV-infected cells by assessing the expression of GFP and Gm44763. (B) Representative plots of GFP expression in LV-Gm44763- or LV-Control-infected CATH.a cells, scale bar = 50 μm. (C) Gm44763 expression was markedly elevated in CATH.a cells transduced with LV-Gm44763. *n* = 6, ****P < 0.0001 compared with the LV-Control group. Student’s *t* test. (D) Representative plots of GFP expression in LV-Gm44763- or LV-Control-infected N2a cells, scale bar = 50 μm. (E) N2a cells infected with LV-Gm44763 exhibited markedly increased expression of Gm44763, significant down-regulation of miR-298-5p, and a concomitant up-regulation of eIF4E mRNA. *n* = 6. *P < 0.05, ****P < 0.0001, compared with the LV-Control, Student’s *t* test. (F) Protein levels of eIF4E were significantly increased in LV-Gm44763-infected N2a cells. *n* = 4. *P < 0.05, compared with the LV-Control-infected group (Student’s *t* test). (G) Flowchart of the rescue experiment for transfection of miR-298-5p mimics/inhibitor in N2a cells infected with LV-Gm44763. (H) The expression of miR-298-5p was significantly increased by transfection with miR-298-5p mimics, and this effect was partially rescued by LV-Gm44763. *n* = 6. **P < 0.01, ****P < 0.0001. (I) The expression of eIF4E was significantly suppressed by transfection with miR-298-5p mimics, and this suppression was partially rescued by LV-Gm44763. *n* = 6. ****P < 0.0001. (J) Transfection with the miR-298-5p inhibitor significantly reduced the expression of miR-298-5p. ****P < 0.0001. (K) miR-298-5p inhibitor transfection significantly up-regulated eIF4E expression, and this effect was further amplified by LV-Gm44763 in a synergistic manner. *n* = 6. *P < 0.05, ****P < 0.0001. Data analyzed using 2-way ANOVA with Bonferroni post hoc test.

To further elucidate the regulatory interactions among Gm44763, miR-298-5p, and eIF4E, we performed regulatory validation experiments in N2a and CATH.a neuronal cell lines. Cells were infected with either LV-Gm44763 or LV-Control. Approximately 72 h post-infection, fluorescence expression and Gm44763 transcript levels were assessed (Fig. 6A). Gm44763 expression in the LV-Gm44763 group was significantly up-regulated by 21.55-fold in Cath cells (Fig. 6B and C). Similarly, in N2a cells, Gm44763 expression was increased by 84.79-fold (Fig. 6D and E). Therefore, subsequent experiments were conducted in N2a cells. The results showed that overexpression of Gm44763 significantly decreased miR-298-5p expression levels (Fig. 6E). Concurrently, eIF4E mRNA and protein levels were significantly up-regulated (Fig. 6E and F). To confirm the direct regulatory effect of miR-298-5p on eIF4E, miR-298-5p mimics or inhibitors were transfected into cells with or without Gm44763 overexpression (Fig. 6G). The results revealed that miR-298-5p mimics suppressed eIF4E expression, which was partially rescued by Gm44763 overexpression (Fig. 6H and I). In contrast, miR-298-5p inhibitors increased eIF4E expression, an effect that was further enhanced by Gm44763 overexpression (Fig. 6J and K).

Collectively, these data support the notion that Gm44763 functions as a molecular sponge that competitively binds miR-298-5p, thereby relieving miR-298-5p-mediated repression of eIF4E. Mechanistically, this ceRNA regulatory network links Gm44763 to translational control via post-transcriptional modulation of eIF4E.

### miR-298-5p bidirectionally modulates the morphine-induced reward memory

**Fig. 7.**
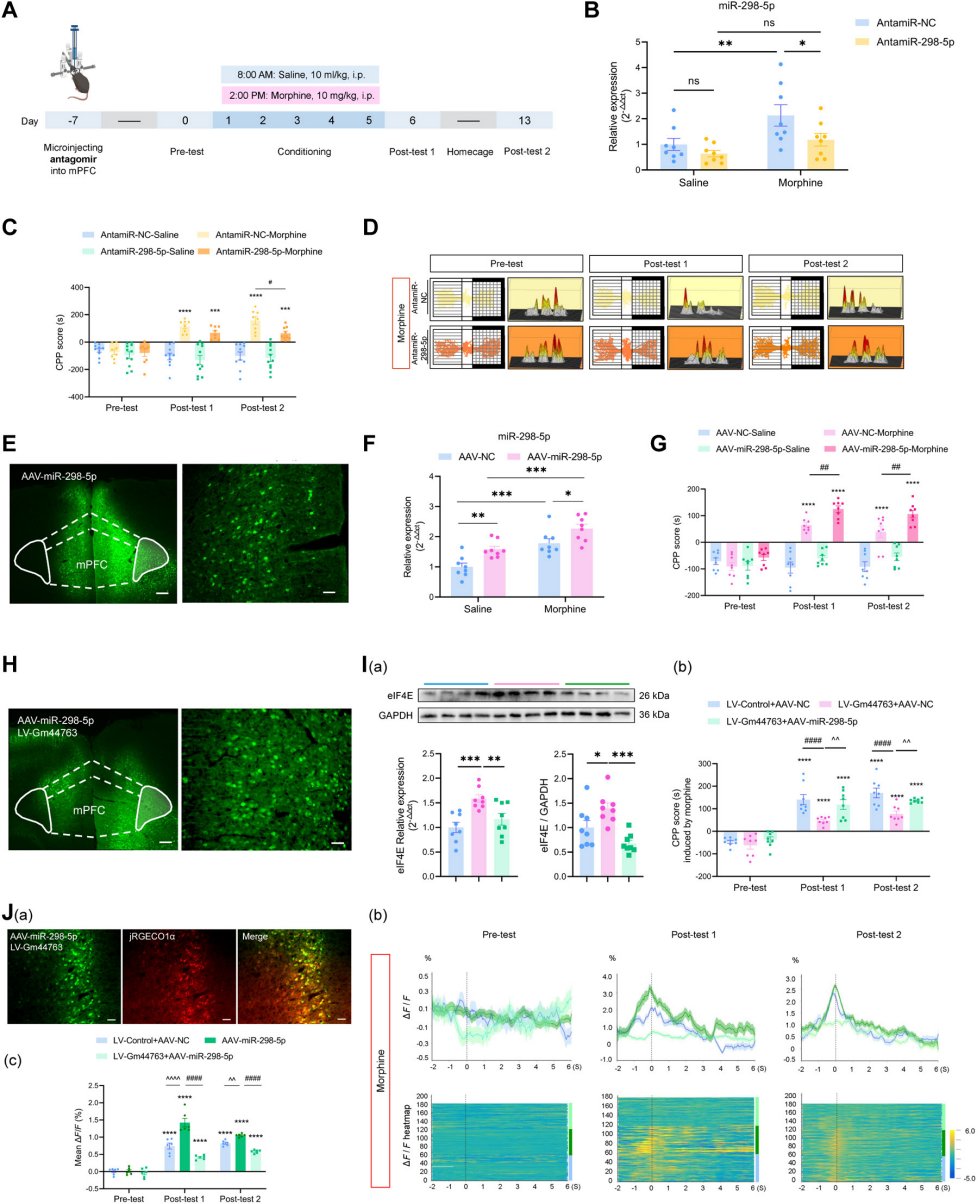
miR-298-5p bidirectionally regulates morphine-induced reward memory and reverses Gm44763-mediated suppression. (A) Schematic timeline of the experiment showing stereotactic delivery of AntagomiR-298-5p into the mPFC 7 days prior to the CPP pre-test. (B) qRT-PCR analysis confirmed that antamiR-298-5p administration specifically reduced miR-298-5p expression in the mPFC of morphine-treated mice. *n* = 8. *P < 0.05, **P < 0.01. (C) AntagomiR-298-5p significantly suppressed morphine-induced CPP scores at post-test 2. *n* = 8 to 10. ***P < 0.001, ****P < 0.0001 compared with pre-test; ^#^*P* < 0.05 compared with antamiR-NC-Morphine. (D) Representative trajectory maps and 3D heatmaps of chamber exploration. (E) Fluorescence validation of AAV-miR-298-5p infection efficiency in the mPFC. Scale bars = 200 μm (left) and 50 μm (right). (F) qPCR analysis confirmed that AAV-miR-298-5p overexpression increased miR-298-5p levels in the mPFC. *n* = 8. *P < 0.05, **P < 0.01, ***P < 0.001. (G) Overexpression of miR-298-5p in the mPFC significantly increased CPP scores during both the post-test 1 and post-test 2. Two-way ANOVA followed by Bonferroni post hoc test, ****P < 0.0001 vs. pre-test score of the same group; ##*P* = 0.006, morphine-induced differences in the LV-Gm44763 group of mice vs. the LV-Control group in post-test 1, ##*P* = 0.002, morphine-induced differences in the LV-Gm44763 group of mice vs. the LV-Control group in post-test 2, *n* = 8. (H) Fluorescence validation of viral transduction efficiency. Scale bars = 200 μm (left) and 50 μm (right). (I) miR-298-5p overexpression reversed the Gm44763-induced increase in CPP scores and eIF4E expression at both mRNA and protein levels. (a) Overexpression of miR-298-5p reversed the significant effects of Gm44763 on both eIF4E mRNA and protein levels. *n* = 8. *P < 0.05, **P < 0.01, ***P < 0.001. (b) Overexpression of miR-298-5p in the mPFC reversed the inhibitory effect of Gm44763 overexpression on reward memory. *n* = 8. ****P < 0.0001 vs. pre-test score of the same group; ####*P* < 0.0001 vs. the control virus-Morphine group; ^^*P* = 0.002 vs. the LV-Gm44763-Morphine group in post-test 1, ^^*P* = 0.009 vs. the LV-Gm44763-Morphine group in post-test 2. Two-way ANOVA followed by Bonferroni post hoc test. (J) AAV-miR-298-5p enhanced morphine-induced neuronal hyperexcitability, which was attenuated by Gm44763 overexpression. (a) Fluorescence confirmed coexpression of the red calcium indicator AAV-hSyn-JRGECO1a and green LV-Gm44763 or AAV-miR-298-5p in the mPFC. Scale bar = 50 μm. (b) Top: Real-time calcium transients upon entry into morphine-paired chambers. Bottom: Event-locked calcium signal heatmap aligned to chamber entry onset (−2 to +6 s). (c) Mean Δ*F*/*F* during chamber entries showed that AAV-miR-298-5p further amplified morphine-induced neuronal hyperexcitability, while concurrent overexpression of Gm44763 significantly attenuated neuronal activity. *n* = 6. ****P < 0.0001 compared with pre-test; ^####^*P* < 0.0001 compared with the AAV-298-5p-Morphine group; ^^*P* < 0.01, ^^^^*P* < 0.0001 compared with the control virus-Morphine group. Data analyzed using 2-way ANOVA with Bonferroni post hoc test.

To investigate the functional role of miR-298-5p in morphine-induced reward memory in mice, we suppressed miR-298-5p expression in the mPFC using antagomir-298-5p (AntamiR-298-5p). The results demonstrated that the inhibitory effect of AntamiR-298-5p persisted for at least 21 days (Fig. S4A). The experimental procedure was conducted according to the timeline shown in Fig. 7A. qPCR analysis performed after behavioral testing confirmed the localized activity of antamiR-298-5p in the mPFC (Fig. 7B). CPP results revealed that inhibition of miR-298-5p impaired both the acquisition and retrieval of morphine-induced reward memory (Fig. 7C). Locomotor trajectories and heatmaps corroborated these findings (Fig. 7D). In addition, antamiR-298-5p had no effect on locomotor activity, confirming the behavioral specificity of the observed effects (Fig. S4B).

In contrast, we employed adeno-associated virus (AAV)-miR-298-5p to specifically overexpress miR-298-5p (Fig. 7E). qPCR analysis of mPFC tissues confirmed the successful overexpression of miR-298-5p (Fig. 7F). Elevated miR-298-5p expression significantly enhanced CPP scores during both the acquisition and retrieval phases of morphine-induced reward memory (Fig. 7G), without affecting locomotor activity (Fig. S4C).

To further investigate the functional interaction between Gm44763 and miR-298-5p in morphine-induced reward memory, we coexpressed LV-Gm44763 and AAV-miR-298-5p in the mPFC (Fig. 7H and Fig. S4D). Two-way analysis of variance (ANOVA) revealed that overexpression of miR-298-5p reversed the significant effects of Gm44763 on both CPP scores and eIF4E mRNA and protein levels (Fig. 7I). These regulatory manipulations did not affect locomotor activity in mice. (Fig. S4E).

We next examined the neural mechanisms that may underlie the behavioral outcomes of the Gm44763/miR-298-5p interaction. We coexpressed the calcium indicator jRGECO1a with either AAV-miR-298-5p alone or in combination with LV-Gm44763 in the mPFC [Fig. 7J(a)] and performed fiber photometry recordings during both the acquisition and retrieval phases of morphine-induced reward memory. The results showed that AAV-miR-298-5p further amplified morphine-induced neuronal hyperactivity. In contrast, coexpression of Gm44763 significantly attenuated neuronal activity (Fig. 7J).

In summary, our results demonstrate that miR-298-5p bidirectionally regulates morphine-induced reward memory. miR-298-5p is capable of reversing both the behavioral and neuronal activity effects mediated by Gm44763. These findings identify miR-298-5p as a key downstream effector of Gm44763 and provide mechanistic evidence that miR-298-5p contributes to the regulation of drug-associated memory by modulating neuronal excitability.

### eIF4E-mediated translational regulation contributes to morphine-induced reward memory

**Fig. 8.**
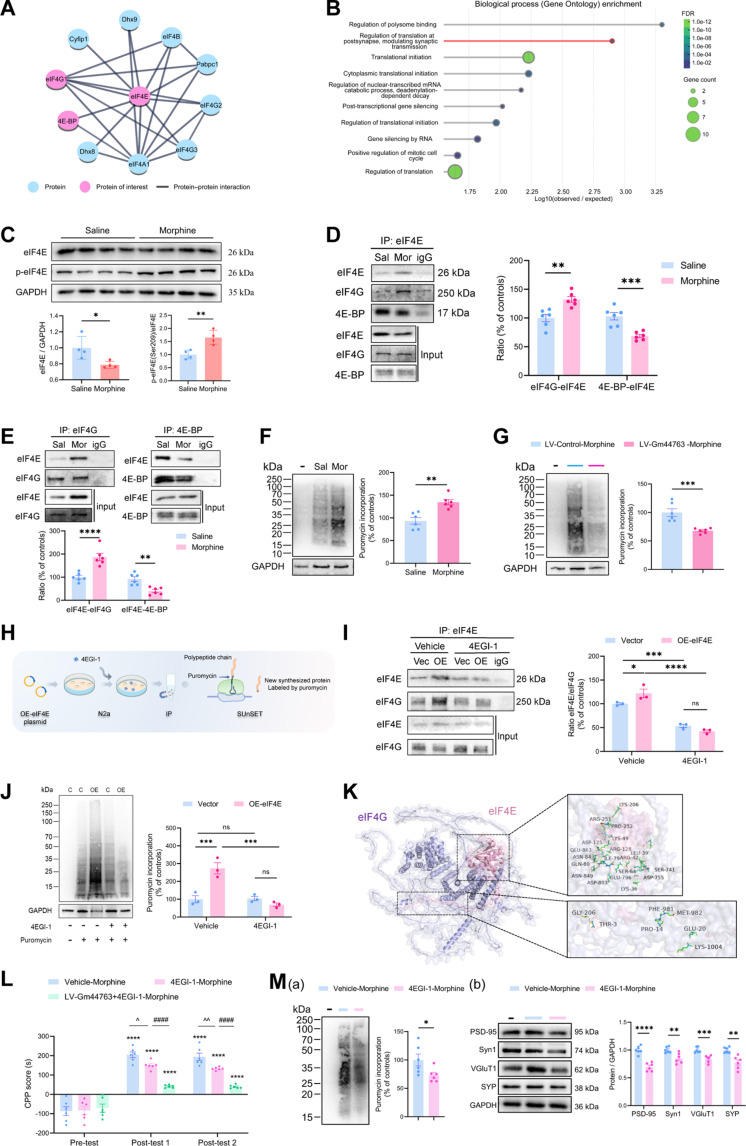
eIF4E-mediated translational regulation is involved in morphine-induced reward memory. (A) eIF4E-mediated PPI network (B) GO enrichment analysis of proteins interacting with eIF4E revealed that the associated biological processes were primarily involved in the regulation of postsynaptic translation and synaptic transmission. (C) Morphine exposure reduces total eIF4E protein levels but increases its phosphorylation at Ser209 in the mPFC. GAPDH was applied as an internal loading control. *n* = 4, *P < 0.05, **P < 0.01 compared with the saline group. Student’s *t* test. (D) IP of eIF4E analysis confirmed that morphine exposure enhances the interaction between eIF4E and eIF4G, while reducing its association with 4E-BP in the mPFC. *n* = 6. **P < 0.01, ***P < 0.001 compared with the saline group, Student’s *t* test. (E) IP analysis using eIF4G (left) and 4E-BP (right) antibodies show that morphine exposure markedly increases the binding of eIF4E to eIF4G and decreases its interaction with 4E-BP in the mPFC. *n* = 6. **P < 0.01, ****P < 0.0001 compared with the saline group, Student’s *t* test. (F) SUnSET assay using puromycin incorporation shows that morphine-treated mice exhibit a significant elevation in overall protein translation in the mPFC. “–“ represents a control sample without puromycin. *n* = 6. **P < 0.01 compared with the saline group, Student’s *t* test. (G) SUnSET assay shows that overexpression of Gm44763 significantly reduced the translation rate in the mPFC compared with the LV-Control group. (Left) Representative images for the puromycin experiment. (Right) Analysis of the effect of Gm44763 on protein synthesis in mPFC. *n* = 6, ***P< 0.001. (H) Schematic workflow of morphine-induced translational activation simulated in N2a cells, inhibited by 4EGI-1, followed by IP and SUnSET assay. (I) 4EGI-1 significantly inhibited the interaction between eIF4E and eIF4G. IP of eIF4E. *n* = 3. *P < 0.05, ***P < 0.001, ****P < 0.0001, 2-way ANOVA followed by Bonferroni post hoc test. (J) Protein synthesis was blocked by 4EGI-1 treatment. *n* = 3. ***P < 0.001. (K) Protein–protein docking analysis revealed the potential interaction model between eIF4E and eIF4G. (L) 4EGI-1 impairs both the formation and retrieval of morphine-induced CPP. Coexpression of Gm44763 further enhances the suppressive effect of 4EGI-1 on CPP scores. *n* = 6. ****P < 0.0001 vs. pre-test; ^*P* = 0.017, ^^*P* = 0.007 vs. Vehicle-Morphine group; ^####^*P* < 0.0001 vs. compared with 4EGI-Morphine group. Data analyzed using 2-way ANOVA with Bonferroni post hoc correction. (M) 4EGI-1 suppresses protein synthesis and synaptic plasticity-related protein expression in the mPFC. (a) Representative results of the SUnSET assay showing reduced global protein synthesis in the mPFC following 4EGI-1 administration. “–“ represents a control sample without puromycin. (b) Western blot revealed concomitant reductions in the expression of synaptic plasticity-related proteins, including PSD-95, Syn1, VGluT1, and SYP, consistent with the overall down-regulation of translation. Data are presented as mean ± SEM, *n* = 6, *P < 0.05, **P < 0.01, ***P < 0.001, ****P < 0.0001, Student’s *t* test.

To further investigate the functional role of the downstream target gene eIF4E within the Gm44763/miR-298-5p regulatory network in morphine-induced reward memory, we first constructed the protein–protein interaction (PPI) network of eIF4E (Fig. 8A). The analysis revealed that eIF4E regulates translation initiation through interactions with either eIF4G or eIF4E-binding protein (4E-BP). Subsequently, GO enrichment analysis was performed on eIF4E-interacting proteins, and the biological process analysis revealed that these proteins are primarily involved in the regulation of postsynaptic translation and synaptic transmission (Fig. 8B), providing further support for the hypothesis that the Gm44763/miR-298-5p/eIF4E axis may modulate morphine-induced reward memory by regulating synaptic homeostasis.

To determine whether eIF4E function is altered in vivo, we examined both total and phosphorylated eIF4E (p-eIF4E) levels in the mPFC of morphine-treated mice. While total eIF4E protein was reduced, its Ser209-phosphorylated form increased (Fig. 8C). Phosphorylation at this site is known to enhance the affinity of eIF4E for eIF4G, thereby promoting the assembly of the translation initiation complex and activating cap-dependent protein synthesis [37,38]. Immunoprecipitation (IP) (Fig. 8D and E) and surface sensing of translation (SUnSET) assays (Fig. 8F) confirmed increased eIF4E–eIF4G interaction and enhanced protein synthesis following morphine exposure. Overexpression of Gm44763 markedly reduced the global translation levels in mPFC of morphine-treated mice (Fig. 8G). This finding provides direct in vivo evidence that Gm44763 negatively regulates protein synthesis in brain regions critical for morphine-induced memory formation.

To validate this mechanism in vitro, we modeled the morphine-induced translational activation in N2a cells (Fig. 8H). Administration of 4EGI-1, a selective inhibitor of eIF4E–eIF4G binding, disrupted their interaction and reduced global protein synthesis (Fig. 8I and J and Fig. S5A). An interaction model further illustrated the binding mechanism between eIF4E and eIF4G (Fig. 8K).

Finally, behavioral experiments demonstrated that administration of 4EGI-1 alone impaired both the formation and retrieval of morphine-induced CPP. Importantly, co-overexpression of Gm44763 potentiated the inhibitory effect of 4EGI-1 on CPP scores without affecting locomotor activity (Fig. 8L and Fig. S5B). Consistent with the in vitro findings that 4EGI-1 inhibits the interaction between eIF4E and eIF4G, 4EGI-1 treatment significantly reduced protein synthesis levels in the mPFC [Fig. 8M(a)]. Furthermore, after SUnSET assays, the expression of several synaptic plasticity-related proteins, including PSD-95, Syn1, VGluT1, and synaptophysin (SYP), was detected on the same blot, and results showed a significant decrease [Fig. 8M(b)]. These results indicate that eIF4E-dependent translational regulation modulates synaptic plasticity in the mPFC during morphine reward memory formation.

These findings reveal a previously unrecognized Gm44763-miR-298-5p-eIF4E regulatory network in the mPFC, providing new insight into the post-transcriptional mechanisms underlying morphine-induced synaptic plasticity and reward memory formation.

## Discussion

**Fig. 9.**
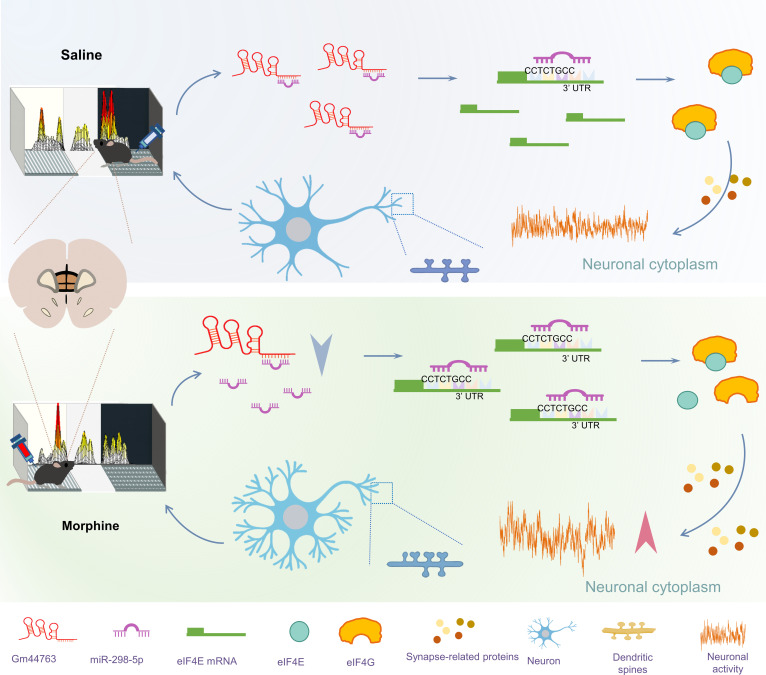
Mechanistic hypothesis diagram. Gm44763 regulates morphine-induced reward memory via the miR-298-5p/eIF4E axis. Down-regulation of Gm44763 following morphine exposure enhances miR-298-5p-mediated suppression of eIF4E, leading to altered synaptic protein synthesis, increased dendritic complexity, and facilitation of reward memory expression.

Chronic opioid abuse leads to alterations in synaptic plasticity, resulting in severe public health consequences. With the development of whole genome sequencing technology, lncRNAs have been increasingly recognized as being associated with substance abuse [39]. Thus, identifying lncRNA-based molecular markers is urgently required for the diagnosis and treatment of morphine exposure. Here, we report for the first time the differential lncRNA expression profile in the mPFC of morphine-induced CPP mice. By integrating our laboratory’s mRNA transcriptomic data uploaded to the GEO database (GSE221797), we constructed a ceRNA regulatory network mediated by DElncRNAs. Notably, this work is the first to demonstrate that the expression of Gm44763 is significantly down-regulated in the mPFC of morphine-induced CPP mice. Furthermore, we proposed the Gm44763/miR-298-5p/eIF4E regulatory axis as a key modulator of drug-associated memory and synaptic plasticity (Fig. 9). These results offer novel insights into the molecular basis of addiction-related memory and point to potential therapeutic targets for opioid addiction.

### Dysregulated lncRNAs are strongly associated with neurological function

In this study, we identified a total of 126 DElncRNAs in the mPFC of morphine-induced CPP model mice, including 22 up-regulated and 104 down-regulated transcripts. Although these DElncRNAs have not been previously reported to be directly associated with substance abuse, accumulating evidence indicates that dysregulated lncRNA expression plays a crucial role in the progression of neurological disorders and addiction. Among the DElncRNAs identified, the most significantly up-regulated transcript was 4930417H01Rik, classified as a processed transcript. Notably, earlier studies reported that 4930417H01Rik expression was increased in mice receiving gut microbiota transplants from patients with major depressive disorder [40], which is consistent with our findings in the morphine reward memory model. This suggests that 4930417H01Rik may be involved in stress-related or reward-associated neuroadaptation. Conversely, the most significantly down-regulated lncRNA was C130073E24Rik, belonging to the class of long intergenic non-coding RNAs. Based on ceRNA network analysis, we further predicted that both 4930417H01Rik and C130073E24Rik may potentially interact with miRNAs such as miR-298-5p and miR-330-5p [41], thereby regulating downstream target genes implicated in neuronal synaptic plasticity. However, it is important to note that these predictions are currently derived solely from bioinformatic analyses and require further validation through comprehensive in vivo and in vitro functional studies to confirm their biological relevance and mechanistic roles.

In addition to the ceRNA mechanism, lncRNAs can also exert cis-regulatory effects by modulating the expression of neighboring protein-coding genes [42]. In this study, we identified that the up-regulated lncRNA Gm28192 is located downstream of the monocarboxylate transporter 4 (MCT4) gene, which is involved in metabolic regulation and cellular signaling within the central nervous system and is believed to play a crucial role in synaptic plasticity and learning and memory processes [43]. Furthermore, the down-regulated lncRNA Gm44763 was identified as an antisense transcript of IQ Motif Containing K (Iqck), a gene that has been implicated as a risk factor for obsessive–compulsive disorder and Alzheimer’s disease in genome-wide association studies [44]. These findings collectively suggest that multiple DElncRNAs identified in this study may be broadly involved in the regulation of neural function and addiction-related behaviors.

In addition, to improve the accuracy of identifying potential ceRNA regulatory networks associated with morphine addiction, we incorporated known genes related to neurological disorders during the selection of target mRNAs and reconstructed multiple ceRNA subnetworks (Fig. S3B and C). Beyond the Gm44763/miR-298-5p/eIF4E axis validated in this study, we identified additional candidate genes with potential functional relevance, including Shisa7, Sod2, and Ptch1. Shisa7 has been demonstrated to function as a regulatory protein for AMPA receptors and is essential for hippocampal synaptic plasticity and memory retrieval [45]. Sod2 single-nucleotide polymorphisms have been reported to be associated with early-onset bipolar disorder [46]. Furthermore, miR-298-5p itself has been implicated in the pathophysiology of neurodegenerative diseases and chronic stress models. Based on these observations, we hypothesize that the 4930417H01Rik/miR-298-5p/mRNA axis potentially involving downstream targets such as Shisa7 and Sod2 may represent an additional ceRNA regulatory pathway contributing to the modulation of morphine-induced reward memory. It is important to emphasize, however, that these conclusions are currently based on bioinformatic predictions. Further functional validation, both in vitro and in vivo, is required to confirm the biological significance of these candidate regulatory networks. Ultimately, such investigations may facilitate the identification of novel molecular targets for the diagnosis and treatment of addiction in clinical settings.

A limitation of the present study is that all experiments were conducted in male mice. Sex differences have been widely reported in addiction-related neurobiology [47]. Therefore, it remains to be determined whether the conclusions of this study operate similarly in females. Future studies incorporating both sexes will be essential to elucidate potential sex-dependent mechanisms in morphine-induced reward memory and the generalizability of our findings.

### Gm44763 functions as a novel modulator in morphine-induced reward memory

In this work, we identified Gm44763 as a novel regulator of morphine-induced reward memory and clarified its function in modulating miR-298-5p and downstream eIF4E via a ceRNA-mediated post-transcriptional pathway. Our experimental findings suggest that Gm44763 may influence the formation and maintenance of drug-associated reward memory by modulating neuronal plasticity and excitability.

Interestingly, Gm44763 exhibited pronounced regional specificity following morphine exposure. Under morphine-conditioned stimulation, Gm44763 was selectively down-regulated in the mPFC, but not in the NAc, BLA, or Hipp. Our results further confirmed that Gm44763 is primarily localized in neurons rather than glial cells, indicating that it functions within neuronal populations. Behavioral analyses additionally demonstrated that modulation of Gm44763 expression did not affect spatial learning, recognition memory, anxiety, or social behaviors, highlighting its specificity for drug-associated reward memory. Together, these findings identify Gm44763 as a selective and functionally specific regulator within the mPFC reward circuitry.

However, the upstream mechanisms by which morphine suppresses Gm44763 remain unclear. Morphine may regulate Gm44763 expression through transcription factors, such as cAMP-response element binding protein (CREB), ΔFosB, and nuclear factor kappa-B (NF-κB), which are known to mediate neuronal plasticity in reward-related circuits and can also regulate lncRNA transcription [48]. In addition, bioinformatic prediction has identified several potential transcription factors, including HMX2 and HNF4A, within the genomic region of miR-298-5p, suggesting that these factors may also indirectly participate in the transcriptional regulation of the Gm44763. By the way, epigenetic mechanisms, including histone modifications, DNA methylation, and chromatin remodeling, may contribute to the down-regulation of Gm44763 [49]. Future studies using approaches such as chromatin immunoprecipitation (ChIP), promoter-reporter assays, or assay for transposase-accessible chromatin using sequencing (ATAC-seq) could help identify the specific transcription factors and epigenetic modifications involved, providing direct evidence for the upstream regulation of Gm44763 by morphine.

It should be noted that while miR-298-5p has been demonstrated in this study to play a role in reward memory regulation, previous research has also highlighted its involvement in other neurological conditions. For instance, miR-298-5p has been reported to induce neuronal apoptosis, a mechanism associated with neurodegenerative disease onset and progression [50]. Moreover, miR-298-5p has been linked to the regulation of depressive-like behaviors in mouse models [32]. Nevertheless, its role in opioid addiction, especially in morphine-induced reward memory formation and retrieval, remains unexplored.

Despite the insights provided by this study into the function of the Gm44763/miR-298-5p/eIF4E axis in morphine reward memory, several limitations should be acknowledged. For instance, while we preliminarily validated the ceRNA regulatory mechanism, it remains unclear whether miR-298-5p also regulates other addiction-related targets, or whether Gm44763 exerts its function through non-ceRNA pathways. Although this study demonstrates that the Gm44763/miR-298-5p/eIF4E axis regulates morphine-induced reward memory, the present findings are limited to the morphine-induced CPP. Whether this molecular pathway also participates in reward memory elicited by other addictive drugs or non-drug reinforcement remains to be determined. Future studies using self-administration or other learning paradigms could help establish whether the observed regulatory mechanism is generalizable across different aspects of addiction. Moreover, this study did not directly investigate the eIF4E–eIF4G interaction. This process may be influenced by several converging mechanisms, such as eIF4E phosphorylation, the availability of binding partners, and synaptic signaling pathways [51,52]. Future studies employing ribosome profiling or IP approaches would help determine whether Gm44763 modulates the eIF4E–eIF4G interaction and identify specific downstream protein targets associated with morphine-induced synaptic plasticity.

### Altered activity state of eIF4E contributes to morphine-induced reward memory

In this study, we further confirmed the critical role of eIF4E as an essential downstream effector within the Gm44763/miR-298-5p regulatory axis in the formation and maintenance of morphine-induced reward memory. Neuronal function relies heavily on eIF4E-dependent translation, which has also been implicated in neurodevelopmental processes and the pathogenesis of several neuropsychiatric disorders [17,53]. The PPI network and GO enrichment analysis revealed extensive interactions between eIF4E and multiple translation regulators, with significant enrichment in biological processes related to postsynaptic translational control and synaptic signaling, suggesting a potential role for eIF4E in reward-related synaptic plasticity mechanisms.

Notably, we observed a significant reduction in total eIF4E protein levels in the mPFC following morphine exposure, accompanied by a marked increase in phosphorylation at Ser209, indicative of enhanced activation. This shift in activity state was paralleled by increased dendritic spine density and heightened neuronal excitability. These phenotypes are consistent with the established role of eIF4E phosphorylation in promoting its affinity for eIF4G, facilitating the assembly of the eIF4F complex and driving cap-dependent translation [38,54]. Our results further confirmed that morphine exposure enhanced eIF4E–eIF4G interactions and up-regulated global protein synthesis. These findings suggest that morphine may activate upstream signaling pathways that convert eIF4E from a translationally quiescent to an active phosphorylated form, thereby promoting the translation of synaptic plasticity-related proteins and facilitating the formation of drug-associated reward memory. A recent study has also identified an alternative cap-dependent translation mechanism in which, under conditions of eIF4E depletion, certain mRNAs can initiate translation via direct interaction between the 5′ cap and eIF3d, bypassing the requirement for eIF4E [55]. Whether this eIF4E-independent mechanism is engaged during morphine-induced plasticity remains to be determined.

We further found that the eIF4E protein level was significantly increased under Gm44763 overexpression condition, but the synaptic structure and neuronal activity were not further enhanced. These findings suggest that the relationship between eIF4E expression, synaptic structural remodeling, and neuronal activity is not strictly linear but may instead involve more complex regulatory mechanisms. The possible reason is that 4E-BP protein plays a key negative regulatory role in this process. As a competitive binding factor of eIF4E, 4E-BP can bind eIF4E with strong affinity in the non-phosphorylated state, thereby inhibiting the overall protein synthesis [56]. In addition, eIF4E-mediated translation is known to exhibit a high degree of selectivity. Previous studies have demonstrated that p-eIF4E preferentially promotes the translation of specific mRNAs in liver tissue [57]. However, whether such selectivity occurs for synaptic plasticity-related transcripts in the mPFC following morphine exposure remains unknown. Future studies employing translatome profiling approaches, such as ribosome profiling sequencing or polysome sequencing, will be instrumental in delineating the scope and specificity of eIF4E-dependent translation in this context.

Moreover, most mechanistic studies of translation initiation have been conducted in non-stressed cells grown in nutrient and oxygen-rich culture conditions. However, many cell types, including tumor cells, frequently experience environmental stress such as hypoxia, nutrient deprivation, or proteotoxic stress, all of which can inactivate mTORC1 and suppress eIF4E activity [58,59]. A similar stress-induced translational repression may occur in morphine-exposed neurons and warrants further investigation. These potential complementary mechanisms do not diminish the central role of the Gm44763/miR-298-5p/eIF4E axis identified in this study. Instead, they highlight the possibility that drug-induced synaptic adaptation is driven by the interplay of multiple converging pathways.

Morphine activates multiple intracellular signaling pathways that underlie reward memory formation, such as the ERK/CREB and phosphatidylinositol 3-kinase (PI3K)/protein kinase B (Akt)/mTOR pathways. Interestingly, these pathways converge on eIF4E [60]. The ERK/MNK1/2 axis phosphorylates eIF4E at Ser209, while mTORC1 activation releases eIF4E from 4E-BP inhibition, and both mechanisms enhance cap-dependent protein synthesis. Our results demonstrate that elevated eIF4E activity facilitates local translation and up-regulates synaptic proteins, thereby promoting reward memory consolidation. Collectively, these findings link the Gm44763/miR-298-5p/eIF4E regulatory axis with the classical signaling networks involved in morphine-induced synaptic plasticity.

In summary, this study identifies the Gm44763/miR-298-5p/eIF4E axis as a critical regulatory pathway underlying morphine-induced reward memory, underscoring the pivotal role of cap-dependent translation in synaptic plasticity and drug-evoked behavioral encoding. While our findings demonstrate that Gm44763 can alleviate morphine-induced synaptic remodeling and CPP behavior via modulation of eIF4E expression and translational activity, several limitations remain. The current investigation is restricted to the mPFC, and the expression profile and functional relevance of Gm44763 in other reward-related brain regions, such as NAc, Hipp, and BLA, have yet to be determined. Moreover, the role of this pathway in critical phases of addiction, including long-term withdrawal and spontaneous relapse, remains unexplored. Future studies are needed to elucidate its dynamic regulatory functions across distinct brain circuits and disease stages.

## Materials and Methods

### Animals

C57BL/6J male mice aged 8 to 10 weeks were obtained by the Experimental Animal Center of Xi’an Jiaotong University. They were housed under standard conditions (12 h light/12 h dark cycle, food and water ad libitum) and acclimated for 1 week prior to experimental manipulations. All experiments were conducted in accordance with the Xi’an Jiaotong University Animal Care Guidelines and approved by the Laboratory Animal Administration Committee (Approval no. 2018-421).

### Drugs

Morphine hydrochloride was obtained from First Pharmaceutical Factory of Shenyang (China) and was given intraperitoneally (i.p.) with a volume of 10 mg/kg. Normal saline (0.9% NaCl) was used as control. All drugs were prepared daily and administered at room temperature.

### CPP

For the CPP study, we randomly divided the mice into 2 groups, morphine and saline infusion group. The CPP apparatus had 3 chambers; chamber color and floor shape provided visual and tactile conditioned cues for mice, respectively. We performed trajectory analysis by Smart 3.0 behavior analysis software (Panlab SL, Barcelona, Spain) to obtain data on the transition, dwell time, and number of shuttles of mice in each chamber. The CPP score was calculated as the difference in time spent in the morphine-paired versus saline-paired compartment on the test day. The protocol was adapted from previously published studies with minor modifications [61].

As shown in Fig. 1A, the CPP procedure comprised 5 phases: the pre-conditioning test (pre-test, day 0), conditioned training period (days 1 to 5), post-conditioning test (post-test 1, day 6), home cage period (days 7 to 12), and post-test 2 (day 13). During the conditioned training period, mice were injected with saline (10 ml/kg, i.p.) or morphine (10 mg/kg, i.p.) and remained in a fixed chamber for 45 min; the chamber naturally preferred by mice during pre-test was used as a non-drug chamber. After returning to the home cage for a week, the mice underwent retrieval test on day 13. For the pre-test and post-tests, mice had free access to both chambers for 15 min.

### Open field test

The OFT was used to evaluate general locomotor activity and anxiety-like behavior in mice. The apparatus consisted of a square open field arena measuring 45 × 45 × 45 cm with opaque walls and uniform illumination. Mice were individually placed in the center of the arena and permitted to explore freely for 10 min. Behavior was recorded using a top-mounted camera and analyzed with an automated video-tracking system (Smart 3.0, Panlab). Key parameters included the distance traveled and time spent in the central and peripheral zones.

### Elevated plus maze

To evaluate anxiety-like behavior in mice, the EPM test was conducted. The maze consisted of 2 open arms (30 × 5 cm) and 2 closed arms (30 × 5 × 15 cm) arranged in a cross configuration and elevated 50 cm above the floor. Mice were placed in the central zone facing an open arm and permitted to freely explore the maze for 5 min under uniform lighting. Behavior was recorded using a top-mounted video camera and analyzed with the SMART 3.0 automated tracking system. The primary parameters measured included the time spent and number of entries in the open arms, closed arms, and the central area.

### Y-maze test

Spatial working memory was assessed using the Y-maze test. The maze comprised 3 identical arms (30 × 6 × 15 cm) positioned at 120° angles to create a Y-shaped structure. One of the arms was designated as the “novel arm” and was blocked during the initial 10-min familiarization phase. After a 2-h intertrial interval, the novel arm was opened, and the mice were allowed to explore all 3 arms freely for 5 min. Mouse behavior was recorded using a top-mounted video camera and analyzed with the SMART 3.0 automated behavioral analysis system. The sequence and total number of arm entries were recorded. Spontaneous alternation was defined as consecutive entries into 3 distinct arms (e.g., ABC and BCA). The alternation percentage was calculated as follows: Alternation (%) = [Number of alternations / (Total arm entries – 2)] × 100.

### NOR task

The NOR test was used to evaluate recognition memory in mice. During the training phase, 2 identical objects were placed in an open field, and each mouse was allowed to explore freely for 5 min. This procedure was repeated 3 times with 15-min intervals between sessions. Three hours after the final training session, one of the familiar objects was replaced with a novel object, and mice were allowed to explore for 5 min (test 1). Twenty-four hours later, the novel object from test 1 was replaced with a different novel object, and the mice were again allowed to explore for 5 min (test 2). The number of interactions with the novel and familiar objects was recorded using a top-mounted camera and analyzed with SMART 3.0 software. The recognition index (RI) was calculated as: RI = [Number of novel object explorations / (Number of novel + familiar object explorations)] × 100%. An RI greater than 50% indicates a preference for the novel object.

### Sucrose preference test

The sucrose preference test (SPT) is a behavioral test that detects an animal’s natural preferences. After viral expression, mice were provided with a 1% sucrose solution for 24 h only to acclimatize the mice to this fluid. For the next 24 h, the mice were free to choose between the 1% sucrose solution and another bottle of water. To prevent the influence of the bottle or its position on drinking behavior, the experimenter changed the position of the bottle after 12 h. The mice were allowed to choose between the 1% sucrose solution and another bottle of water. Then, water was forbidden for 24 h. On the fourth day, the consumption of water and sucrose solution was calculated by weighing the bottles and calculating the sucrose consumption rate as: Sucrose preference = Sucrose consumption (g) / [Sucrose consumption (g) + Water consumption (g)] × 100%.

### RNA sequencing

Immediately after CPP post-test, mice were executed and mPFC brain regions were isolated for RNA-seq (*n* = 3 samples per group). RNA-seq technology mainly includes 3 experiments. Firstly, RNA quantification and qualification were performed. High-quality RNA-seq reads were obtained after filtering, and rRNA was removed using the Epicentre Ribo-zero Removal Kit (Epicentre, WI, USA), followed by ethanol precipitation to eliminate residual rRNA. LncRNA libraries were then generated from rRNA-depleted RNA using the NEBNext Ultra Directional RNA Library Prep Kit for Illumina (CA, USA), according to the manufacturer’s protocol. Finally, library clustering was performed on the cBot Cluster Generation System using the TruSeq PE Cluster Kit v3-cBot-HS (Illumina). Sequencing was then carried out on the Illumina HiSeq 4000 platform to generate 150-bp paired-end reads. Raw sequencing data have been deposited in the GEO database (GSE221797).

### Differential lncRNA expression analysis

We used StringTie (v1.3.3) to calculate the FPKMs of lncRNAs in each sample. We added the FPKMs of each transcript to calculate the gene FPKMs. FPKM refers to the fragment mapped per million fragments per kilobase exon, which is calculated according to the length of the fragment and the read count mapped to the fragment. The differential expression of lncRNA in the morphine group and saline group was analyzed by the DEGseq R package (1.28.0). |Fold change| > 1.5 and *P* < 0.05 were set by default as the threshold for significant differential expression.

### GO and KEGG enrichment analysis

GO enrichment analysis of DEmRNAs cis-regulated by DElncRNAs was implemented by the clusterProfiler R package (v3.12.0). *P* < 0.05 GO terms were considered significantly enriched. KEGG enrichment analysis was performed using KEGG Orthology Based Annotation System (KOBAS) (v3.0) [62]. *P* < 0.05 KEGG pathways were considered significantly enriched.

### Construction of the lncRNA–miRNA–mRNA ceRNA regulatory network

The ceRNA network is based on the theory that lncRNAs can interact with miRNAs to play a sponge role to regulate mRNAs activity. As shown in Fig. S3A, we utilized miRDB and RNAhybrid2.2 databases to predict target miRNAs of differential lncRNAs. Next, we used miRWalk3.0, TargetScan v7.2, ENCORI, and RNAhybrid2.2 databases to predict the target miRNA of DEmRNA and select the binding site at 3′ UTR [63]. Subsequently, we integrated the interaction among lncRNAs, miRNAs, and mRNAs to establish the ceRNA regulatory network. CeRNA subnetworks containing mRNAs related to neurological related diseases was also established. The ceRNA networks were visualized with the Cytoscape software (version 3.10).

### Quantitative real-time PCR

Total RNA was isolated using the RNAfast1000 Total RNA Extraction Kit (Pioneer Biotechnology, Shaanxi, China) following the manufacturer’s instructions. RNA concentration was detected using NanoDrop 2000c (Thermo Fisher Scientific, CA, USA). RNA (500 ng) was reverse transcribed into cDNA with Hifair III 1st Strand cDNA Synthesis SuperMix for qPCR (Yeasen Biotechnology, Shanghai, China) or miRNA 1st strand cDNA synthesis kit (Accurate Biology, Hunan, China). Then, we analyzed qPCR reaction using the SYBR Green Premix Pro Taq HS qPCR Kit (Accurate Biology). Relative expression of lncRNA, mRNA, and miRNA was calculated using the 2^−ΔΔCq^ method, with GAPDH or U6 used as the internal control to normalize the data. The information of lncRNA-, miRNA-, and mRNA-related primers is shown in Tables S2 to S4.

### FISH assay

RNA FISH assay was performed according to the manufacturer’s instructions using a FISH kit (BersinBio, Guangzhou, China) to observe the subcellular localization of Gm44763 in CATH.a and N2a cells and mPFC brain slices. BersinBio designed and synthesized Cy3-labeled Gm44763 probe and FAM-labeled miR-298-5p probe. Briefly, cell crawls or tissue slices were fixed with 4% formaldehyde for 15 min at room temperature. Cells were then permeabilized with 20 μg/ml proteinase K working solution. Afterwards, cells were incubated with the probe overnight at 42 °C for hybridization. DAPI (4′,6-diamidino-2-phenylindole) staining of nuclei was performed for 5 min. Fluorescence images were obtained by fluorescence microscopy (Leica, Hessian, Germany).

### FISH combined with immunofluorescence staining

After hybridization of mPFC sections with a specific probe against Gm44763, immunofluorescence staining was performed using antibodies targeting neuronal and glial markers. The following primary antibodies were used: anti-NeuN (Proteintech, 26975-1-AP, 1:100), anti-Iba1 (MedChemExpress, YA353, 1:50), and anti-S100β (Abways, CY5201, 1:50). Appropriate fluorophore-conjugated secondary antibodies were applied for visualization. Fluorescence signals were acquired using a high-resolution fluorescence microscope to assess the cell-type-specific distribution of Gm44763.

### LV and AAV stereotactic injection in the mPFC

The mice were anesthetized with 5% isoflurane and fixed in the brain stereotaxic apparatus. The anterior and posterior bregma of mice were exposed. The fontanel bregma was the origin, and the Hamilton micro syringe was slowly lowered to the mPFC injection site (AP: +1.98 mm, ML: ±0.30 mm, DV: −2.2 mm). The viruses involved in the experiment are shown in Table 1. The needle was left in place for 10 min after injection before being withdrawn slowly. Behavioral experiments were performed after confirming adequate viral expression levels.

**Table 1.** Summary of virus

Virus ID	Viral vector	Titer (TU/ml)	Manufacturer
LV-Gm44763	Ubi-MCS (Gm44763)-CBh-gcGFP-IRES-puromycin	4E+8	Genechem (Shanghai, China)
LV-Control	Ubi-MCS (Control)-CBh-gcGFP-IRES-puromycin	5E+8	Genechem (Shanghai, China)
AAV-miR-298-5p	AAV9-CMV bGlobin-EGFP-MCS (miR-298-5p)-WPRE-hGH polyA	1E+12	Genechem (Shanghai, China)
AAV-NC	AAV9-CMV bGlobin-EGFP-MCS (NC)-WPRE-hGH polyA	1E+12	Genechem (Shanghai, China)
JRGECO1a	pAAV-hSyn-NES-JRGECO1a-WPRE	2.11E+13	OBiO (Shanghai, China)

### Golgi–Cox staining

Mouse brains were harvested and fixed, followed by conventional dehydration, paraffin embedding, and sectioning. Brain sections were then immersed in Golgi–Cox impregnation solution, which was refreshed every 3 days. Utilizing the argyrophilic properties of neurons, the neuronal structures in the brain tissue were selectively stained black. After staining, sections were rinsed with distilled water, incubated overnight in 80% glacial acetic acid, and then cryoprotected in 30% sucrose solution before sectioning. Sections were mounted and sealed with neutral balsam under light-protected conditions. Neuronal morphology and dendritic spine density were quantified using ImageJ software. Dendritic complexity was assessed by Sholl analysis, with concentric circles drawn at 10-μm intervals from the soma to measure the number of dendritic intersections.

### Fiber photometry for calcium dynamics

Real-time calcium dynamics in the mPFC were recorded using fiber photometry. Mice were anesthetized with isoflurane, and the red fluorescent calcium indicator jRGECO1a along with experimental viral constructs were stereotaxically injected into the mPFC. An optical fiber cannula (400 μm core diameter, 0.39 NA, 3 mm length; RWD Life Science, China) was implanted targeting the same region. After recovery, fiber photometry was performed during the CPP tests. A fluorescence detection system (R820, RWD, China) was used, with 560 nm excitation for jRGECO1a and 410 nm as an isosbestic control. Photometric signals were acquired in parallel with behavioral data during the CPP tests. Data were processed using a multi-channel fiber photometry software suite. Calcium signals were quantified by calculating the relative fluorescence change (Δ*F*/*F*) to evaluate neuronal activity during the acquisition and retrieval phases of morphine-induced reward memory.

### RNA immunoprecipitation

RIP assays were conducted in N2a cells using the BersinBio RNA-Binding Protein Immunoprecipitation Kit (BersinBio) following the manufacturer’s instructions. Briefly, 2 × 10^7^ cells were collected, washed, and lysed in 900 μl of RIP lysis buffer. Lysates were incubated overnight at 4 °C with 4 μg of anti-Ago2 (ab186733, Abcam, Cambridge, UK) or control IgG (BersinBio), followed by incubation with 40 μl of protein A/G magnetic beads for 2 h at 4 °C. RNA was then isolated by phenol–chloroform extraction, and Gm44763, miR-298-5p, and eIF4E levels were quantified by qPCR.

### Dual-luciferase reporter assay

Dual-luciferase reporter assay was employed to assess the direct binding of miR-298-5p to Gm44763 and eIF4E. Bioinformatics analyses were conducted to predict the binding sites of miR-298-5p with Gm44763 and eIF4E. 293T cells were cotransfected with either the wild-type reporter plasmid pmirGLO-WT or the mutant reporter plasmid pmirGLO-MUT, along with miR-298-5p mimics or a mimic negative control (mimics NC). Twenty-four hours post-transfection, Firefly luciferase levels were measured using a dual luciferase reporter kit and normalized to Renilla luciferase activity.

### Cell culture

Neuro-2a (N2a) and CATH.a cells were purchased from Procell (Wuhan, China) and maintained and stored according to the instructions provided by the supplier. Briefly, cells were maintained in Dulbecco’s modified Eagle medium (DMEM) with 1% penicillin and streptomycin and 10% fetal bovine serum. Cells were incubated at 37°C in a humidified incubator containing 5% CO_2_. All cells were subjected to STR identification and mycoplasma contamination assay.

### Cell transfection

Cells were transfected using Lipofectamine 2000 (Invitrogen, CA, USA) according to the manufacturer’s instructions. Briefly, N2a cells were cultured to 80% fusion in 24-well plates. Subsequently, the synthetic miR-298-5p mimics/inhibitor, siRNA eIF4E, and its corresponding control were transfected into N2a cells with Lipofectamine 2000 reagent according to the manufacturer’s instructions. After 48 h of transfection, cells were harvested for subsequent experiments. Sequences of the above constructs are shown in Table 2.

**Table 2.** Sequence used in cell transfection

Definition	Sequence (5′–3′)
miR-298-5p-mimics	GGCAGAGGAGGGCUGUUCUUCCC
NC-mimics	UCACAACCUCCUAGAAAGAGUAGA
miR-298-5p-inhibitor	GGGAAGAACAGCCCUCCUCUGCC
NC-inhibitor	UCUACUCUUUCUAGGAGGUUGUGA

### Western blot

Western blot was performed according to the protocol developed. Briefly, tissue and cellular proteins were extracted using RIPA lysate (strong), and the proteins were denatured in a metal bath at 95 °C for 5 min. Samples were separated on sodium dodecyl sulfate-polyacrylamide gel electrophoresis (SDS-PAGE) and then transferred to the nitrocellulose membrane. Primary antibodies were diluted according to the recommended ratio (anti-eIF4E, Proteintech Group 66655-1-Ig, 1:15,000; anti-PSD-95, Proteintech Group 20665-1-AP, 1:2,000; anti-VGluT1, Synaptic Systems 135302, 1:20,000; anti-Syn1, Proteintech Group, 30549-1-AP, 1:5,000; anti-SYP Proteintech Group, 17785-1-AP, 1:5,000; anti-p-eIF4E, abways CY5419, 1:1,000) and overnight at 4 °C. Secondary antibodies (1:5,000) were incubated on a shaker at room temperature for 2 h. The ECL chemiluminescence kit was used to detect the bands.

### Immunoprecipitation

Cells were lysed and subjected to IP with a commercial kit following the manufacturer’s instructions. Primary antibodies included anti-eIF4E (Santa Cruz Biotechnology, sc9976g, 3 μg), anti-eIF4G (Proteintech, 15704-1-AP, 3 μg), and anti-4E-BP (Huabio, ET1701-83, 3 μg). The antibody–lysate mixtures were gently rotated overnight at 4 °C. Normal IgG was used as a negative control. The next day, Protein A/G-conjugated magnetic beads were added and incubated to allow antibody–bead binding. After thorough washing of the magnetic beads to remove unbound material, the bound proteins were eluted from the beads. Both the eluates and wash fractions were collected for downstream analysis to evaluate the efficiency of IP. The band intensity of eIF4E was normalized to the amount of eIF4G immunoprecipitated in the same sample to assess interaction strength.

### SUnSET assay for protein synthesis

To assess global protein synthesis, the SUnSET was employed [64]. Cells were incubated with 10 μg/ml puromycin under standard culture conditions for 15 min to allow incorporation of puromycin into nascent polypeptide chains. After treatment, cells were harvested and lysed on ice using RIPA buffer supplemented with protease inhibitors. Equal amounts of total protein (50 μg) were separated on a 4% to 20% SDS-PAGE gradient gel, followed by transfer to polyvinylidene difluoride (PVDF) membranes. Puromycin-labeled polypeptides were detected using an anti-puromycin antibody (Sigma-Aldrich ZMS1016, 1:10,000) and visualized by enhanced chemiluminescence with a chemiluminescence imaging system.

### Molecular docking

Mouse eIF4E and eIF4G protein structures were retrieved from UniProt (https://www.uniprot.org/), which provides curated information on protein sequences and functions reported in the literature. Rigid-body molecular docking was performed using the Global RAnge Molecular Matching (GRAMM) web server (https://gramm.compbio.ku.edu/) to assess the potential interaction between the 2 proteins. In the docking procedure, eIF4E was designated as the receptor and eIF4G was assigned as the ligand. The top-ranked model from the GRAMM output was selected as the final model. Molecular visualization of the docking interface was performed using PyMOL (Schrödinger, LLC, USA).

### Statistical analysis

Data are presented as mean ± SEM. For normally distributed data with equal variances, 2-group comparisons were analyzed using an unpaired *t* test. One-way ANOVA was applied for single-factor comparisons across groups, and 2-way ANOVA for 2-factor analyses, followed by Bonferroni’s post hoc test. Exact *n*, statistical tests, and multiple-comparison corrections are reported in each figure legend. Statistical analysis used SPSS 27.0 statistics software and GraphPad Prism 9.0.0 software. *P* < 0.05 was considered statistically significant.

## Data Availability

The data that support the findings of this study are available from the corresponding authors upon reasonable request. All sequencing data have been deposited at NCBI GEO (GSE221797).
