# Supplementary material for: Novel LncRNA Gm44763 Regulates Morphine-Induced Reward Memory via MiR-298-5p-Mediated eIF4E Translation Control
Source: Research (Wash D C). 2026 Jan 8;9:1032. doi: 10.34133/research.1032 (PMC12779893; doi:10.34133/research.1032)
Supplement: Supplementary 1 — Figs. S1 to S5 Tables S1 to S4 [file research.1032.f1.docx]

**Supporting Information for**

**Novel lncRNA Gm44763 Regulates Morphine-induced Reward Memory via miR-298-5p-mediated eIF4E Translation Control**

*Feifei Gao^1,2†^, Xixi Yang^1,2†^, Zhuojin Yang^1,2^, Dongyu Yu^1,2^, Bao Zhang^1,2^, Yihan Wang^3^, Zhen Yao^4^, Jie Chen^1,2^, Qi Liao^4^, Lanjiang Li^5^, Beilin Hou^1,2^, Danmei Wang^1,2^, Yuxiang Zhang^1,2*^, Chunxia Yan^1,2,4*^*

**This PDF file includes:**

Table S1 to S4

Figures S1 to S5

**Supplementary Tables**

| **Table S1. The detailed information of top 10 up-regulated and 10 down-regulated lncRNAs** | | | |  |
| --- | --- | --- | --- | --- |
| **DElncRNAs** | **log2FoldChange** | | **P-value** |  |
| 4930417H01Rik | 1.00972613 | | 0.027009708 |  |
| Gm20402 | 0.838555596 | | 0.038118195 |  |
| Gm48249 | 0.756288375 | | 0.006575385 |  |
| Gm16214 | 0.735493749 | | 0.040908393 |  |
| Gm16104 | 0.733338333 | | 0.028604698 |  |
| Gm30934 | 0.731318932 | | 0.018351199 |  |
| 1700110K17Rik | 0.721176766 | | 0.003317884 |  |
| Gm42413 | 0.681819966 | | 0.000312837 |  |
| Gm28192 | 0.656467704 | | 0.024243409 |  |
| 4930524O07Rik | 0.638510413 | | 0.008939583 |  |
| C130073E24Rik | -2.165108632 | | 0.025763611 |  |
| Gm44763 | -1.64797821 | | 0.008564277 |  |
| Gm48357 | -0.896672465 | | 0.000957199 |  |
| Gm26971 | -0.895932191 | | 0.018595852 |  |
| Gm43847 | -0.816342446 | | 3.23472E-07 |  |
| Gm47405 | -0.800738459 | | 0.003774119 |  |
| Gm42854 | -0.780745938 | | 0.013547197 |  |
| Gm15802 | -0.737166299 | | 0.020082679 |  |
| Gm17116 | -0.729810725 | | 0.047858707 |  |
| Gm15397 | -0.719679759 | | 0.025819467 |  |
| **Table S2. Information of lncRNA primers** | | | | |
| **Gene** | | **Primer Sequence** | | |
| 1700110K17Rik | | F: GACGGAAGAGGATTTGGG | | |
|  |  | R: GCTGGAGCACTGGGATTT | | |
| Gm28192 | | F: GCGGCTGTGGCTGGATT | | |
|  |  | R: GCTTGGTGTTGGGCTTTC | | |
| Gm30934 | | F: AGACGGCACAGGCAGATAG | | |
|  |  | R: GTGGATTGGCGGGAGTT | | |
| Gm16214 | | F: GGTGGGGGAAGGAGAGAGAT | | |
|  |  | R: TATCTCAGCCAGGGACACGA | | |
| Gm44763 | | F: CAGGGTGCTGTTAGTGGATGAA | | |
|  |  | R: GGGCAGAGACTAGCAGTAAAGAGG | | |
| Gm42854 | | F: GACCGAGGCTCACATGACG | | |
|  |  | R: GGGCTCTGGGAAGAACACTT | | |
| Gm26971 | | F: GCTGCTTCTGTTTCACCTGC | | |
|  |  | R: GCAATCGGGCACTTTTCCAG | | |
| Gm47405 | | F: GGAGCATCCACCCAGCAT | | |
|  |  | R: ACCACCCCTTTCCCACA | | |

| **Table S3. Information of miRNA primers** | |
| --- | --- |
| **Gene** | **Primer Sequence** |
| miR-298-5p | ACACTCCAGCTGGGGGCAGAGGAGGG |
| miR-370-3p | GCCTGCTGGGGTGGAACCTGGT |

| **Table S4. Information of mRNA primers** | |
| --- | --- |
| **Gene** | **Primer Sequence** |
| eIF4E | F: ACCCCTACCACTAATCCCCC |
|  | R: CAATCGAAGGTTTGCTTGCCA |
| Nrxn1 | F: AACGGACTGATGCTTCACACA |
|  | R: CCTGAGTGCTGACGCAGATT |

**Supplementary Figures**

**Fig. S1**


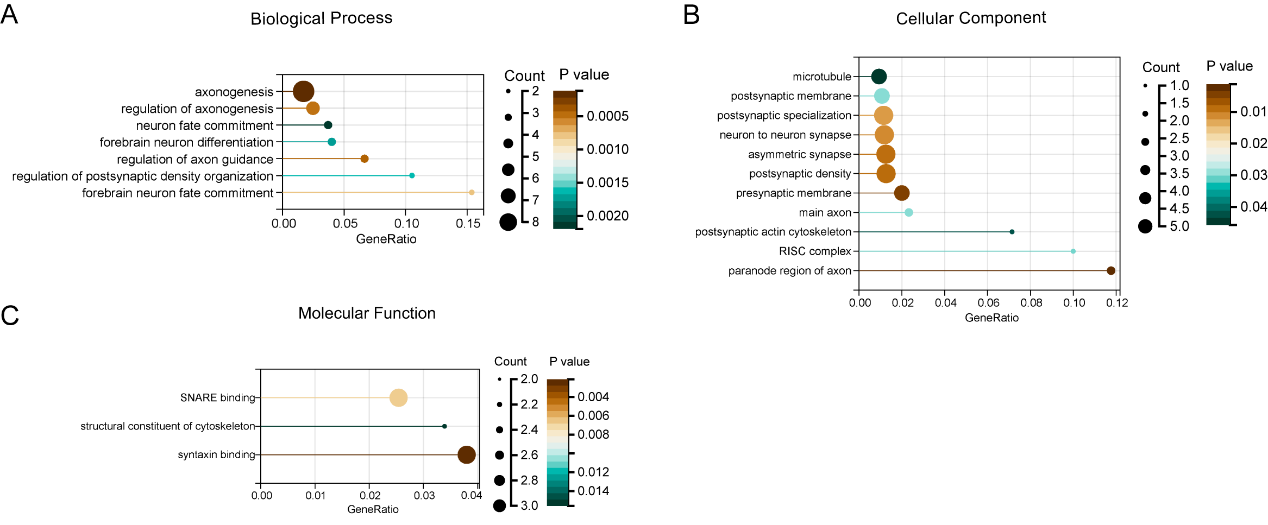


**Fig. S1** GO enrichment analysis of DEmRNAs. Only terms related to synaptic plasticity are shown. (A) Biological processes analysis revealed that DEmRNAs were significantly associated with the processes of axonogenesis (GO:0007409), regulation of axonogenesis (GO:0050770), and regulation of axon guidance (GO:1902667). (B) Cellular components analysis revealed significant enrichment of synapse-related terms including postsynaptic density (GO:0014069), asymmetric synapse (GO:0032279), neuron to neuron synapse (GO:0098984), and postsynaptic specialization (GO:0099572). (C) Molecular functions analysis indicated that syntaxin binding (GO:0019905), SNARE binding (GO:0000149) and structural constituent of cytoskeleton (GO:0005200) were significantly enriched.

**Fig. S2**


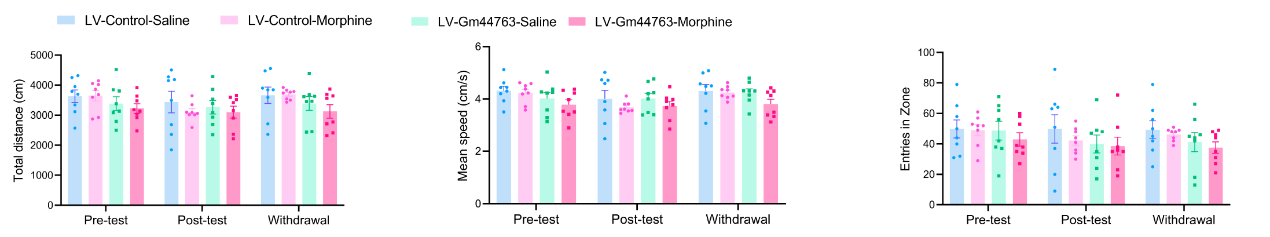


**Fig. S2** Overexpression of Gm44763 did not alter locomotor activity in mice. No significant differences were observed between groups in total distance traveled, mean speed, or shuttle times across the Pre-test, Post-test 1 and Post-test 2. n = 8, two-way ANOVA followed by Bonferroni post-hoc test.

**Fig. S3**


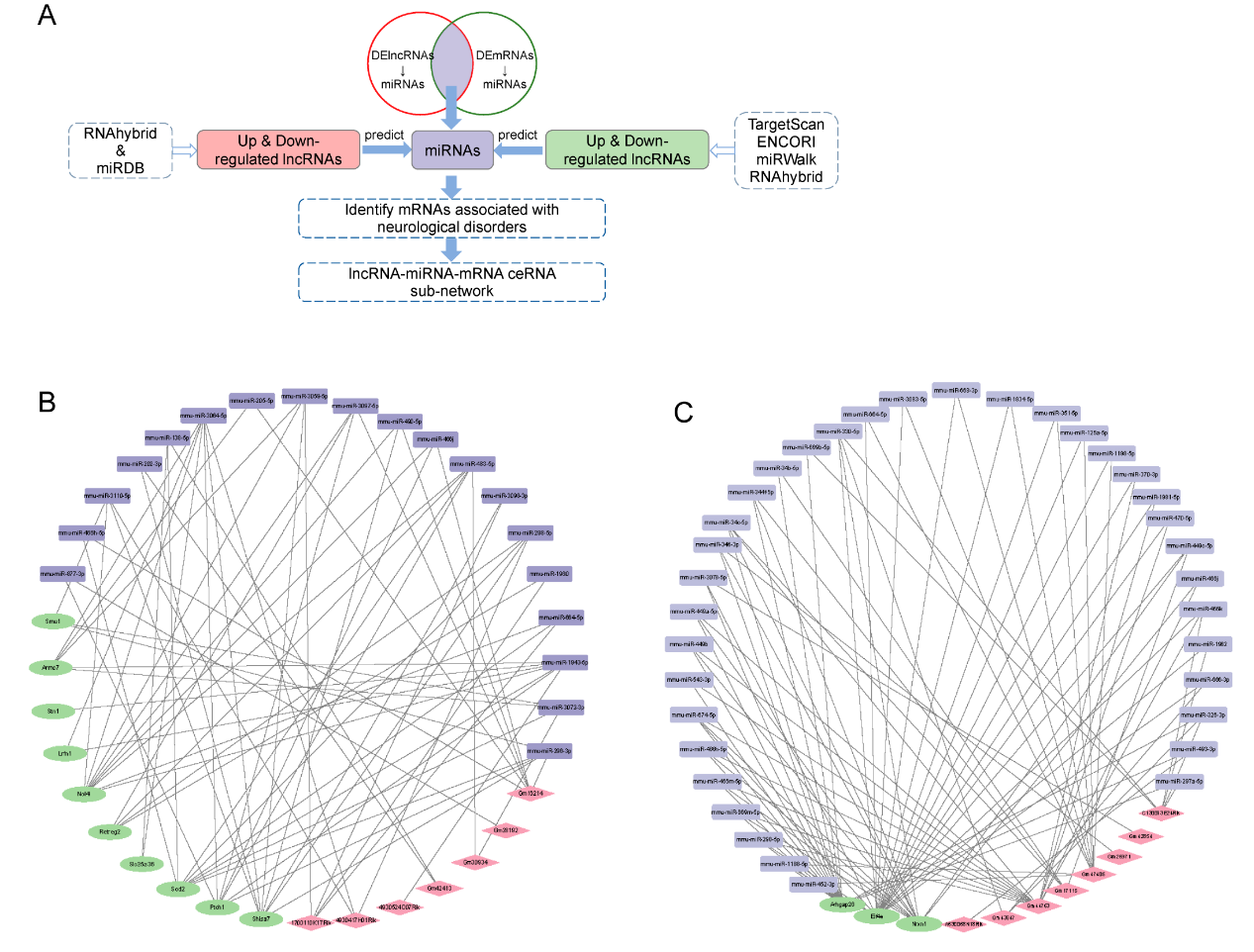


**Fig. S3** Construction of a lncRNA-mediated ceRNA network (A) Flowchart of the ceRNA analysis. (B-C) The nervous system disease associated lncRNA-miRNA-mRNA ceRNA network. Red Diamonds represent lncRNAs, purple rectangles represent miRNAs, and green ellipses represent mRNAs. (B) This network included upregulated lncRNAs and upregulated mRNAs. (C) The network included downregulated lncRNAs and downregulated mRNAs.

**Fig. S4**


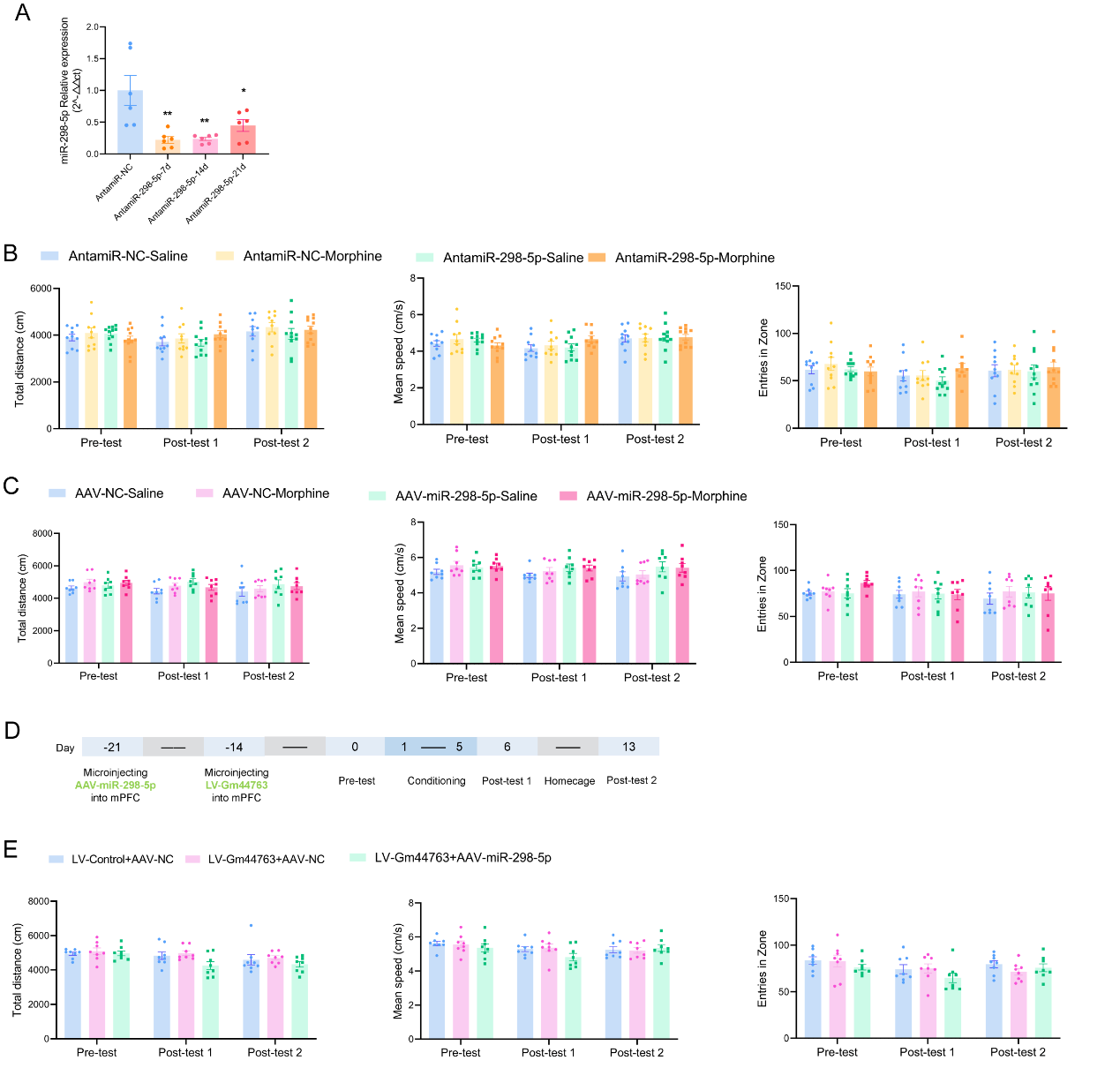


**Fig. S4** Bidirectional modulation of miR-298-5p did not affect locomotor parameters associated with morphine-induced CPP. (A) After antagomiR-298-5p injection, mPFC tissues were collected on days 7, 14, and 21. qPCR analysis confirmed that the inhibitory effect on miR-298-5p expression was maintained for at least 21 days. n=6. **P* < 0.05, ***P* < 0.01, compared to antamiR-NC group. One-way ANOVA followed by Bonferroni post-hoc test. (B) AntagomiR-298-5p did not affect locomotor activity in mice. n = 10-11. (C) AAV-miR-298-5p did not affect locomotor activity in mice. n = 8. (D) Schematic timeline of the experiment showing stereotactic delivery of AAV-miR-298-5p into the mPFC 21 days prior to the CPP pre-test, followed by injection of LV-Gm44763 into the mPFC one week later. (E) Co-expression of AAV-miR-298-5p and LV-Gm44763 had no effect on locomotor activity in mice. n = 8.

**Fig. S5**


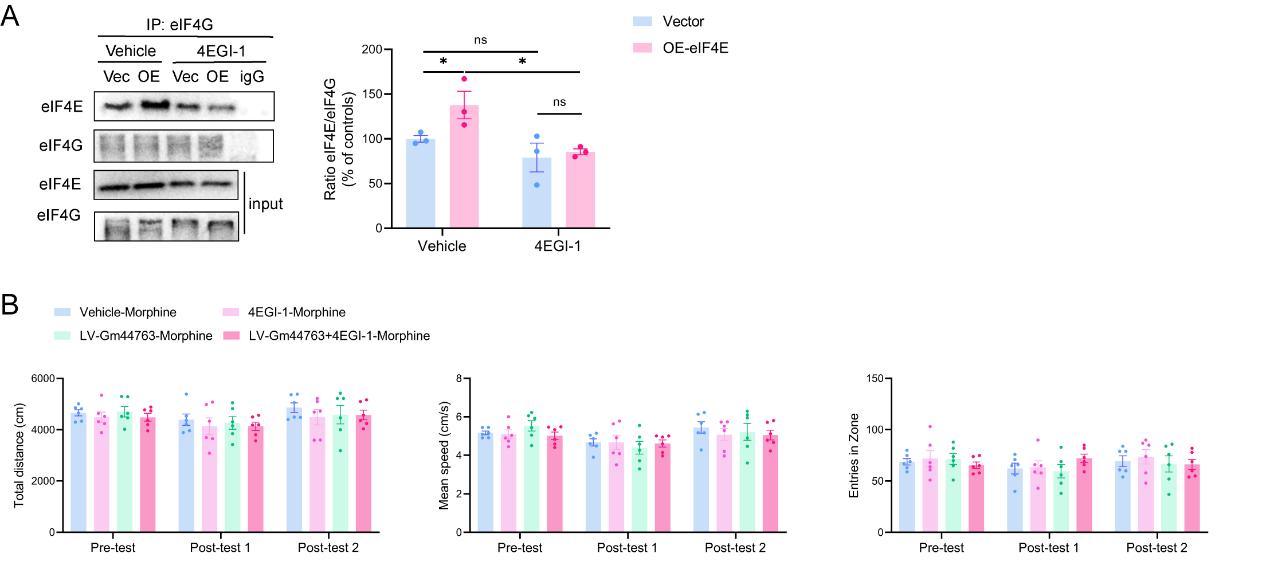


**Fig. S5** (A) IP of eIF4G showed the eIF4E–eIF4G interactions are blocked following treatment of 4EGI-1. n = 3. **P* < 0.05, two-way ANOVA followed by Bonferroni post-hoc test. (B) Injection of 4EGI-1 into the mPFC had no effect on locomotor activity in mice. No significant differences were observed between groups in total distance traveled, mean speed, or shuttle times across the Pre-test, Post-test 1 and Post-test 2. n = 6, two-way ANOVA followed by Bonferroni post-hoc test.
